# The effect of prediction error on episodic memory encoding is modulated by the outcome of the predictions

**DOI:** 10.1038/s41539-023-00166-x

**Published:** 2023-05-29

**Authors:** Francesco Pupillo, Javier Ortiz-Tudela, Rasmus Bruckner, Yee Lee Shing

**Affiliations:** 1 Department of Psychology, Goethe University Frankfurt, Frankfurt, Germany; 2 TS Social and Behavioral Sciences, Tilburg University, Tilburg, Netherlands; 3 Department of Education and Psychology, Freie Universität Berlin, Berlin, Germany; 4 Max Planck Research Group NeuroCode, Max Planck Institute for Human Development, Berlin, Germany

**Keywords:** Learning algorithms, Human behaviour

## Abstract

Expectations can lead to prediction errors of varying degrees depending on the extent to which the information encountered in the environment conforms with prior knowledge. While there is strong evidence on the computationally specific effects of such prediction errors on learning, relatively less evidence is available regarding their effects on episodic memory. Here, we had participants work on a task in which they learned context/object-category associations of different strengths based on the outcomes of their predictions. We then used a reinforcement learning model to derive subject-specific trial-to-trial estimates of prediction error at encoding and link it to subsequent recognition memory. Results showed that model-derived prediction errors at encoding influenced subsequent memory as a function of the outcome of participants’ predictions (correct vs. incorrect). When participants correctly predicted the object category, stronger prediction errors (as a consequence of weak expectations) led to enhanced memory. In contrast, when participants incorrectly predicted the object category, stronger prediction errors (as a consequence of strong expectations) led to impaired memory. These results highlight the important moderating role of choice outcome that may be related to interactions between the hippocampal and striatal dopaminergic systems.

## Introduction

In our daily interaction with the environment, we are confronted with a massive amount of information that cannot all be processed in detail, given the limited resources of our cognitive systems. In order to simplify the complexity of incoming information, our brain tries to extract its regularities to be able to react to environmental demands in efficient ways. One way of extracting regularities from experiences is to rely on repetitions and associations of events, which lead to the creation of increasingly complex knowledge^1,2^. The accumulation of knowledge across similar experiences that can subsequently form expectations and orient our actions is one of the aspects that characterize reinforcement learning^3^. In reinforcement learning, individuals incrementally learn, over experiences, to form expectations in order to successfully predict future events. This learning typically depends on the history of rewards associated with a particular action^4^. However, in real life learning does not always involve such explicit rewards. As an example, we may learn after several experiences that bookstores tend to specialize in different genres, such as crime or romance. Learning this association will allow us to anticipate the type of books in a given bookstore and thus guide us on which one to visit if we are looking for a particular book.

Events can, however, sometimes deviate from our expectations. We may go to a bookstore we have learnt being specialized in the crime genre and discover that the crime novel we are looking for is not there. We might then decide to go to another bookstore, which we know has broad coverage of different genres. Despite a low expectation, we may surprisingly find the crime novel we are looking for in that generalist bookstore. In such situations, the difference between expectations and the experienced event generates a prediction error (PE) signal. The PE is crucial in promoting learning by driving the updating of prior knowledge and changing future expectations and decisions^5,6^. The amount of PE is determined by the strength of the expectations and by the outcome of the predictions. Correctly predicting an event generally results in an increase in the strength of the expectations that led to the prediction, whereas incorrectly predicting leads to a weakening of the related expectations^4^. For example, correctly predicting that the book we are looking for is in a specific bookstore will increase our belief that we need to go to that store to find similar books. Conversely, when we do not find the book in the store our belief will not be as strong as before.

The study of the relationship between prior expectations and learning has benefited from the use of computational models. Reinforcement learning models, in particular, have been used, due to their ability to confer a precise mechanistic role to PE in learning and map it to its neural substrates. It has been shown that firing patterns of mesencephalic dopamine neurons and also Blood-Oxygen-Level Dependent (BOLD) signal change in the striatum resemble the PE signal used in reinforcement learning models^7–9^. This dopamine-dependent PE is thought to inform future predictions by indicating deviations between observed and predicted outcomes^4,10–12^, thus encouraging the repetition of actions that are better than expected (positive PE) and discouraging the repetition of actions that are worse than expected (negative PE)^13,14^.

In addition to incrementally learning from multiple episodes, individuals are also capable of forming episodic memory of distinct, temporally specific events. For example, remembering the precise occasion on which the desired book was found in a store. While there is a great amount of evidence on the effects of PE on incremental learning, its effects on the formation of new episodic memories are still not fully understood. An emerging line of research examines the relationship between prediction errors and episodic memory formation as one form of interaction between memory and learning. These studies are motivated by both anatomical considerations that the hippocampus, the brain structure responsible for the creation of new memories, receives dopaminergic input^15^, and by functional findings showing interactions between the hippocampus and striatum^16–18^. Moreover, recent evidence suggests that hippocampus activation is modulated by the strength of the predictions and their violations^19,20^.

Several studies have manipulated the amount of the reward participants expected and received, linking the obtained reward PE experienced at the time of item presentation or immediately after presentation to the subsequent episodic recognition of those items^21–25^. Some studies have found improved memory for surprising outcomes, namely better memory for items associated with both better- and worse-than-expected outcomes, that is, unsigned PE^21,22^. By contrast, other studies found that better-than-expected outcomes (positive PE), compared to worse-than-expected ones (negative PE), led to improved later recognition^23–25^. Therefore, mixed evidence has been gathered concerning the effects of reward PE on episodic memory, depending on the sign of PE.

The aforementioned studies using computational models manipulated expectations by using rewards. Since in everyday life, learning does not occur always in the presence of explicit rewards, it is crucial to consider the mechanistic effects of PE per se, in contexts in which no external reward is involved. A different way of looking at the relationship between expectations and memory may involve generating them through associations between a context (e.g., going to a bookstore to look for a book) triggering some expectations (e.g., more or less strong belief about the presence of the book), and a matched or unmatched outcome (e.g., finding or not finding the book). In addition, the studies cited above have looked at the effects of PE in conditions in which participants were actively learning novel associations. However, in everyday life, familiar situations in which the environmental structure has been somewhat internalized and expectations that are already established are more common.

Therefore, the present study pre-trained participants to form expectations of varying strengths, which were subsequently matched or mismatched to render PE of different strengths and related these to episodic memory performance. Specifically, we designed a task in which participants learned probabilistic associations between contexts and object categories. After a learning phase in which the expectations were established, participants were presented with the contexts and had to predict the category of trial-unique objects that would appear at the end of each trial. In order to generate different levels of PE strength, we quantified expectations by using gradually different contingencies. The probability of a certain category following a context was systematically manipulated so that for some contexts expectations were stronger than for others. Because no explicit rewards were employed, we do not refer to signed/unsigned PEs.

Findings reported in a separate publication using the same data as the current study^26^ showed that such context manipulation affected recognition memory. Specifically, memory was found to be better for contexts characterized by weaker expectations, compared to contexts in which expectations were stronger. In the present study, we fitted computational models to the data from the learning and encoding phases to perform PE-based memory analyses. More specifically, we used a reinforcement learning model to derive learning rates and trial-level PE experienced during the presentation of unique object images (i.e., encoding phase) and related them to the likelihood of subsequently recognizing the items in a following surprise recognition memory test. The computationally derived PE reflected in a quantitative and gradual manner how unexpected the presentation of an object category in a given context was to the participants. The procedure thus allowed us to test whether PE and prediction outcome at the moment of the presentation of the items were linked to episodic memory encoding.

We reasoned that if unpredicted events per se improve memory encoding, we would observe a positive relationship between PE and memory encoding, so that memory performance would be better for the more unpredicted events. Contrarily, if the effect of PE on memory is modulated based on whether or not the prediction was correct, we would observe an interaction between PE and the choice outcome so that PE improves memory for better-than-expected outcomes, while it impairs memory for worse-than-expected outcomes. To test these hypotheses, we had participants learn associations between context and object categories. The learned associations were then used to predict the occurrence of new objects. Our results showed that the likelihood of correctly recognizing an item scaled with PE and was modulated by prediction outcome at encoding. Specifically, when participants correctly predicted the object category, stronger PE during encoding led to improved recognition in a subsequent memory test, whereas for incorrect predictions stronger PE led to impaired encoding. Together, these results suggest that the strength of memory encoding critically depends on whether or not prior expectations about encoded episodes are correct, in line with the idea that encoding is more successful after more positive PE.

## Results

### Learning performance

**Fig. 1:**
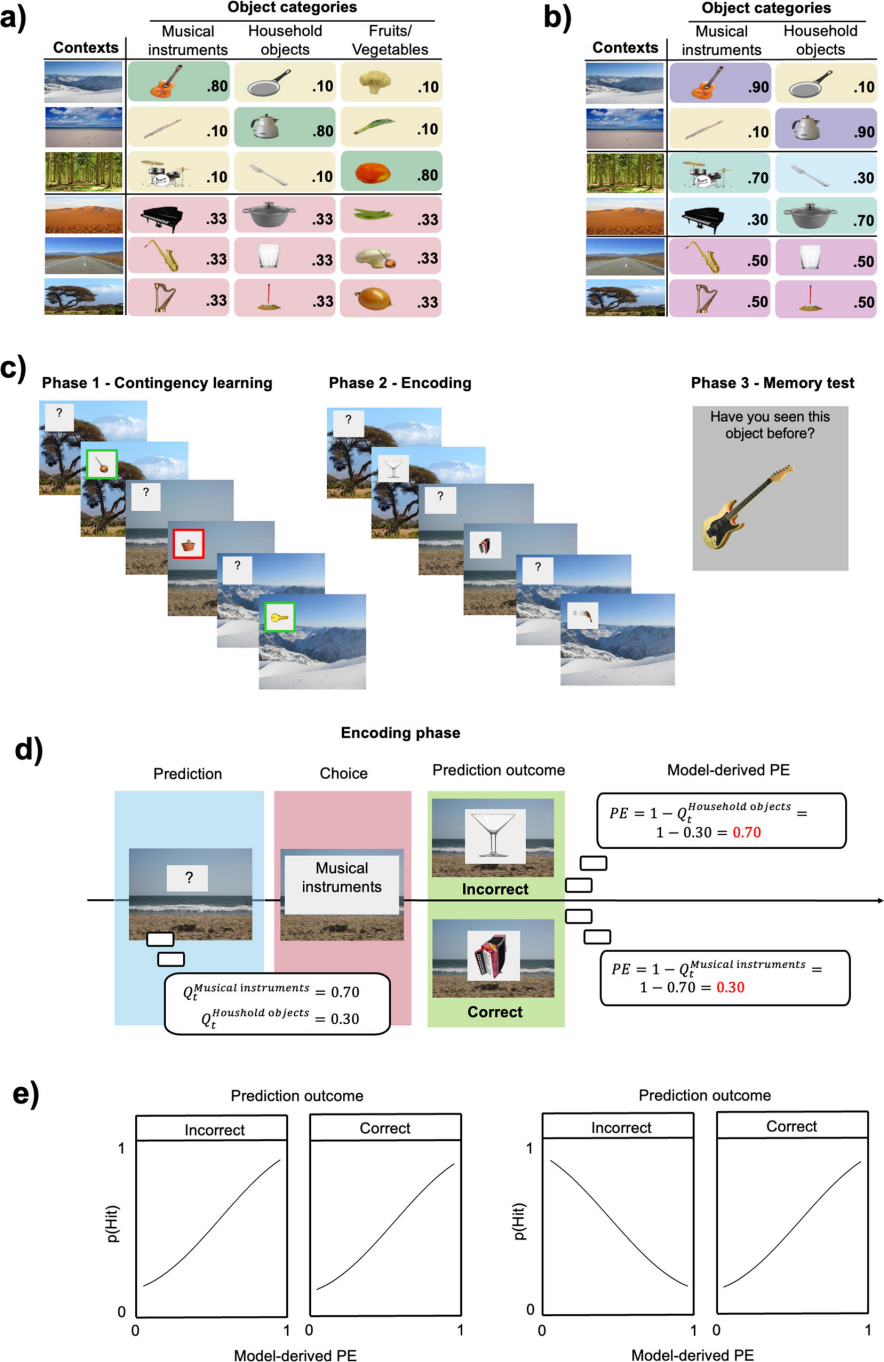
Illustration of the methods. Illustration of the context/object-category contingencies for **a** Experiment 1 and **b** Experiment 2. Note that each object is representative of its object category and that different, unique items from each category were used on every trial. **c** Illustration of the three phases of the study. **d** The model computed different expected values *Q* at each trial. Participants’ category choices could be correct or incorrect. The PE depended on the expected value of the category presented at the end of each trial. This model-derived PE at encoding was then related to the probability of subsequently recognizing those objects among distractors (p(hit)) in a logistic regression model. **e** Two hypothetical relationships between PE and recognition memory. On the left, positive relationship between PE and recognition memory, regardless of prediction outcome. On the right, the prediction outcome modulates the PE-memory interplay.

In both experiments, participants first performed a task in which they were asked to predict the category of trial-unique objects that would follow a specific context. Instructions were given to indicate that each context was predictive of one object category, but there was no indication about which category and with which contingency (see Fig. 1a–c). Participants learned the associations between contexts and object categories during a learning phase (phase 1), in which they received feedback on every trial. In a subsequent encoding phase (phase 2), a new set of never-seen-before objects (but belonging to the same object categories as the ones in phase 1) was introduced and participants were asked to continue doing the same task as in the previous phase. Finally, they completed a recognition memory test, where they were asked to recognize the objects presented in phase 2, among distractors that were never seen before and should be rejected.

In both experiments, participants were presented with six contexts. Each of the contexts was predictive of the object categories following specific contingencies (Fig. 1a, b). In Experiment 1, in half of the contexts one of the three object categories was presented 80% of the time, and the remaining two object categories 10% of the time. In the other half of the contexts, all three object categories were equally likely. In Experiment 2, we included a contingency condition in which the most likely object category was slightly less dominant (0.70–0.30), a manipulation that allowed us to sample more points along the PE continuum. In addition, two instead of three object categories were used, in order to decrease the number of objects for each category and the number of scene contexts per contingency condition needed to enable proper counterbalancing. As a result, the object-context contingencies were 0.90–0.10, 0.50–0.50, and 0.70–0.30, respectively.

**Fig. 2:**
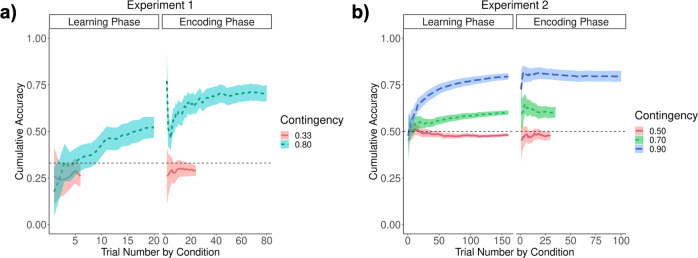
Learning performance. Participants’ performance at the learning and encoding phase for **a** Experiment 1 and **b** Experiment 2. Trial number by contingency condition is represented in the *x*-axis, while cumulative accuracy is shown on the *y* axis. The different colours represent the contingency conditions. The shadows represent the standard error of the mean while the black horizontal dashed lines indicate the chance level. Note that the number of trials differs across conditions, as filler objects were used for stronger contingency conditions, compared to weaker ones to reach the desired contingency.

In both Experiments 1 and 2, learning performance during the contingency learning and encoding phases showed that participants understood the task correctly and were able to learn to predict the object category that was more likely to be presented for each context. Participants’ cumulative accuracy showed that participants clearly favoured the most likely option for each context and experiment (other than the context with equal contingencies), as shown in Fig. 2a, b.

### Computational models

To get a better mechanistic understanding of the pattern observed, we fitted a computational model to participants’ learning data to derive trial-by-trial PE at encoding and link it to subsequent recognition memory (see Fig. 1d, e). In order to derive learning rate and trial-level PE for the contingency learning and encoding phases, variants of a reinforcement learning model^27^ were fitted to data from both the learning and encoding phases pooled together. The use of reinforcement learning models allowed us to capture the process of establishing prior expectations while learning the object-category contingencies of the different contexts. In reinforcement learning models, an agent is assumed to learn values of context–category associations by adding the current expected value to the PE multiplied by a learning rate *α*. The learning rate *α* takes on a value between 0 and 1 and determines the influence of the current PE on the expected values. It represents the extent to which evidence from the current trial is used to update the expectations: Higher learning rates weight the PE more strongly to make the expected values look more like the currently observed one, while lower learning rates weight past estimates more strongly.

We fitted four different reinforcement learning models that made different assumptions on how participants learned the context/object-category associations (see the “Methods” section). These models can be distinguished depending on how the learning rate is estimated and on the type of feedback they use to update the values. For the learning rate, we considered both models assuming the same learning rate for all participants, and models in which the learning rate was free to vary. For the feedback used to update the values, we considered “instructive” models which updated the expected values depending on the object category presented on each trial, regardless of participants’ choices. After having established that the model with a free, constant learning rate fitted the data the best, we also considered an “evaluative” model, which updated the expected values depending on the accuracy of participants’ actions.

Overall, the fitted models were: (a) an instructive model with a learning rate *α* (fixed across participants) that decreases across trials (dLRI), and updates the expected values by increasing the value of the object category presented on a given trial and decreasing the values of the categories not presented, regardless of the choices made by the participants; (b) an instructive model with a decreasing learning rate *α* that was free to vary between individuals (dfLRI); (c) an instructive model with a free constant learning rate *α* (fLRI); and (d) and an evaluative model with a free constant learning rate (fLRE), where the expected values were updated depending on the accuracy of participants’ actions, increasing for correct predictions and decreasing for incorrect ones.

The dLRI considers how the expected values should be updated optimally since it is derived from a Bayesian formulation of the task (see the “Methods” section and Supplemental Material). In this optimal Bayesian formulation of learning, PE is assumed to have its maximal influence on learning in the early trials and decreases as a function of the number of trials. In this model, the only parameter that was estimated was the inverse temperature *β*, which regulates the stochasticity/determinism trade-off in selecting the action depending on the expected values: Higher values of *β* represent a more probable preference for the higher context/object-category associations, while lower values also consider low-strength associations, producing more noisy choices.

In addition to the *β* parameter, the instructive model with the free decreasing learning rate estimates a learning rate *α* that was free to vary across individuals and decreased across the number of observed trials. The instructive fLRI and evaluative fLRE models also estimated a learning rate *α* that was free to vary across individuals, in order to capture participants’ distinct learning rates. However, in contrast to the dLRI and the dfLRI models, in the fLRI and fLRE models, the learning rate *α* was constant throughout the learning and encoding phases. These models make different assumptions on how participants used the PE to update the expected values. In fact, while the evaluative free-learning rate model (fLRE) assumes that participants use the feedback received (correct vs. incorrect) to update only the category chosen, the instructive free-learning rate model (fLRI) implies that on each trial participants update all the associations by strengthening the one between the context and the category presented while lowering the associations with that context and the categories that were not presented at that trial.

**Fig. 3:**
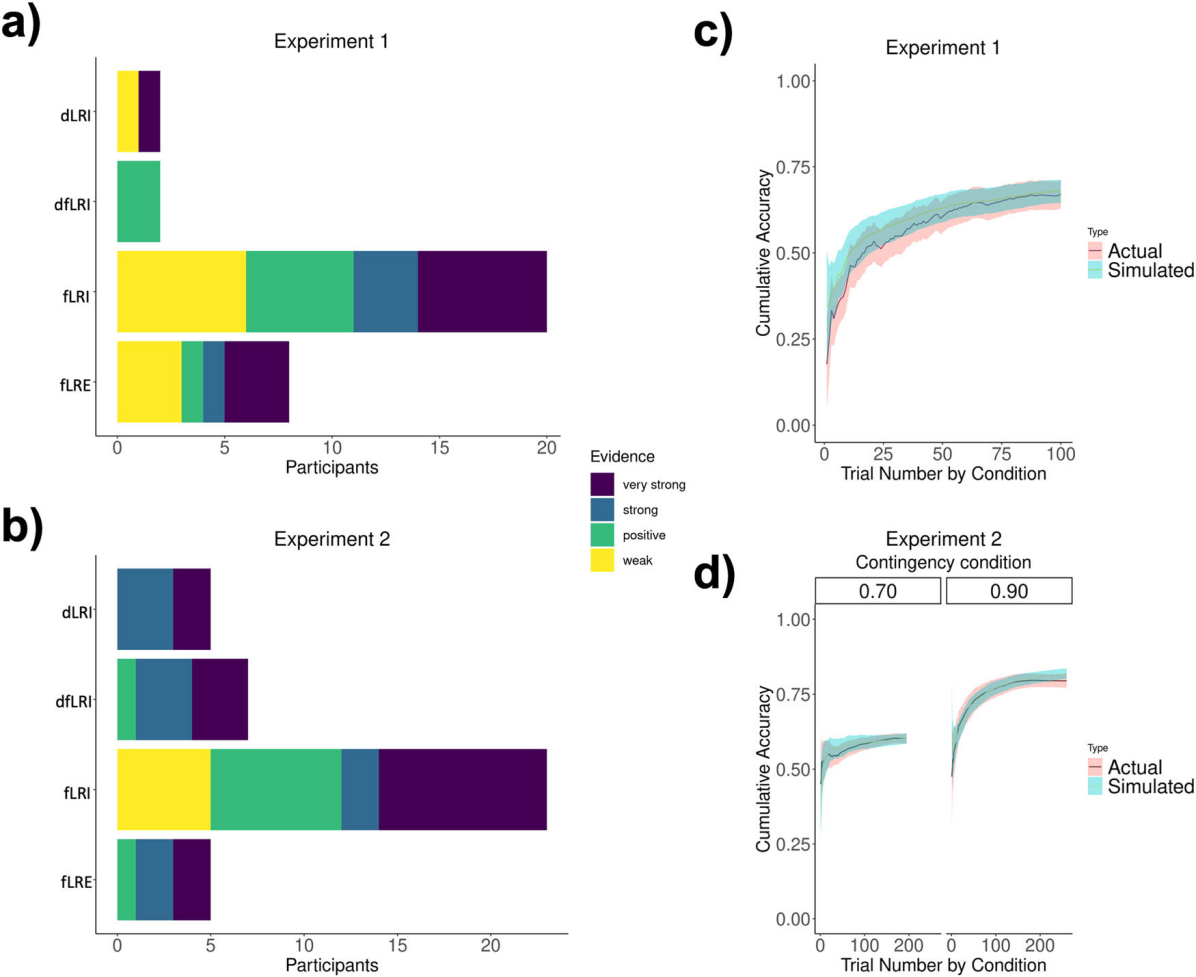
Model comparison and model validation. Results of model comparison for **a** Experiment 1 and **b** Experiment 2. Evidence strength for the best model for each participant is shown. Simulated data of the best fitting model (fLRI) and actual participants’ data overlaid, for **c** Experiment 1 and **d** Experiment 2.

Prior to fitting the models to participants’ data, we ensured that the models could distinguish among different parameter values and also generate qualitatively different data (see the 'Parameter recovery’ and 'Model recovery’ subsections in the “Methods” section and Supplemental Material). Then, the four models were fitted to participants’ data so that the parameters of best fit were estimated as the parameters that maximized the likelihood of participants’ choices. In addition to calculating the models’ log-likelihoods, we calculated the Bayesian information criterion (BIC) for each model and for each subject, by multiplying the maximum likelihood (i.e., the likelihood for the parameters of best fit) by the number of free parameters in the model. This approach penalizes models with more parameters. We then marked the number of participants for which each model was the best fitting, as well as the evidence for it, computed as the BIC difference between the best and the second-best model. Results are shown in Fig. 3a, b. Table 1 shows BIC values and the number of participants for which a model was the best fit, as well as the number of participants for which there was strong evidence, for both Experiments 1 and 2.

**Table 1:** Model comparison.

Model/Experiment	BIC (se)	Best (*N*)	Very strong (*N*)
*Experiment 1*			
dLRI	289.3 (2.5)	2	1
dfLRI	277.0 (2.0)	2	0
fLRI	266.4 (1.7)	20	6
fLRE	271.4 (2.7)	8	3
*Experiment 2*			
dLRI	801.2 (4.3)	5	2
dfLRI	793.1 (2.9)	7	3
fLRI	783.1 (3.7)	23	9
fLRE	774.3 (2.9)	5	2

Model comparison established that the instructive model with the free, constant learning rate (fLRI) explained participants’ behaviour better than the other models. The overall BIC of fLRI was the smallest (indicating better fit), and the number of participants for which it was the best model was 20 over a total of 32 participants for Experiment 1 and 23 over a total of 40 participants for Experiment 2. In addition, there was very strong evidence for it being the best model for 6 participants in Experiment 1 and 9 participants in Experiment 2. These results indicated that participants’ learning processes overall deviate from the behaviour of an optimal Bayesian observer and that they can be better described by using individual learning rates that are constant across trials. In addition, the model comparison showed that most participants used the category information of the object presented at the end of each trial to update all the context/object-category associations, and not only the associations related to the chosen object category.

We then validated the winning model by looking at the ability of the best-fitting model to generate performance that was qualitatively similar to participants’ actual behaviour. Figure 3c, d shows that the model is able to capture participants’ behaviour. Data simulated from the dLRI, dfLRI, and the fLRE models can be found in the Supplemental Material (Fig. S5). We validated the winning model further by looking at the ability of the model to capture differences in learning rates. Simulations showed that the model generated performance that was qualitatively similar to participants’ actual behaviour. The results of this comparison can be found in the Supplemental Material (Fig. S6).

### Model-derived PE and memory

We ran the fLRI model with the best-fitting parameters over the participant data to obtain an estimate of their trial-level PE during the encoding phase. Figure 1d shows how model-derived PE was computed. Participants’ expected values for each object category were computed on each trial and used to derive trial-level PE. The best-fitting model estimated PE by subtracting the expected value of the presented category from 1. The PE estimated was thus unsigned and contingent on the presented category. Consequently, higher PE levels were generated when a category presented was not expected, as reflected by its corresponding expected value being smaller. By contrast, lower PE was generated on trials in which the category presented was characterized by a high expected value. The PE derived at encoding was then linked to the likelihood of successfully recognizing the unique objects as “old” in the subsequent recognition memory test by using a logistic regression model.

**Fig. 4:**
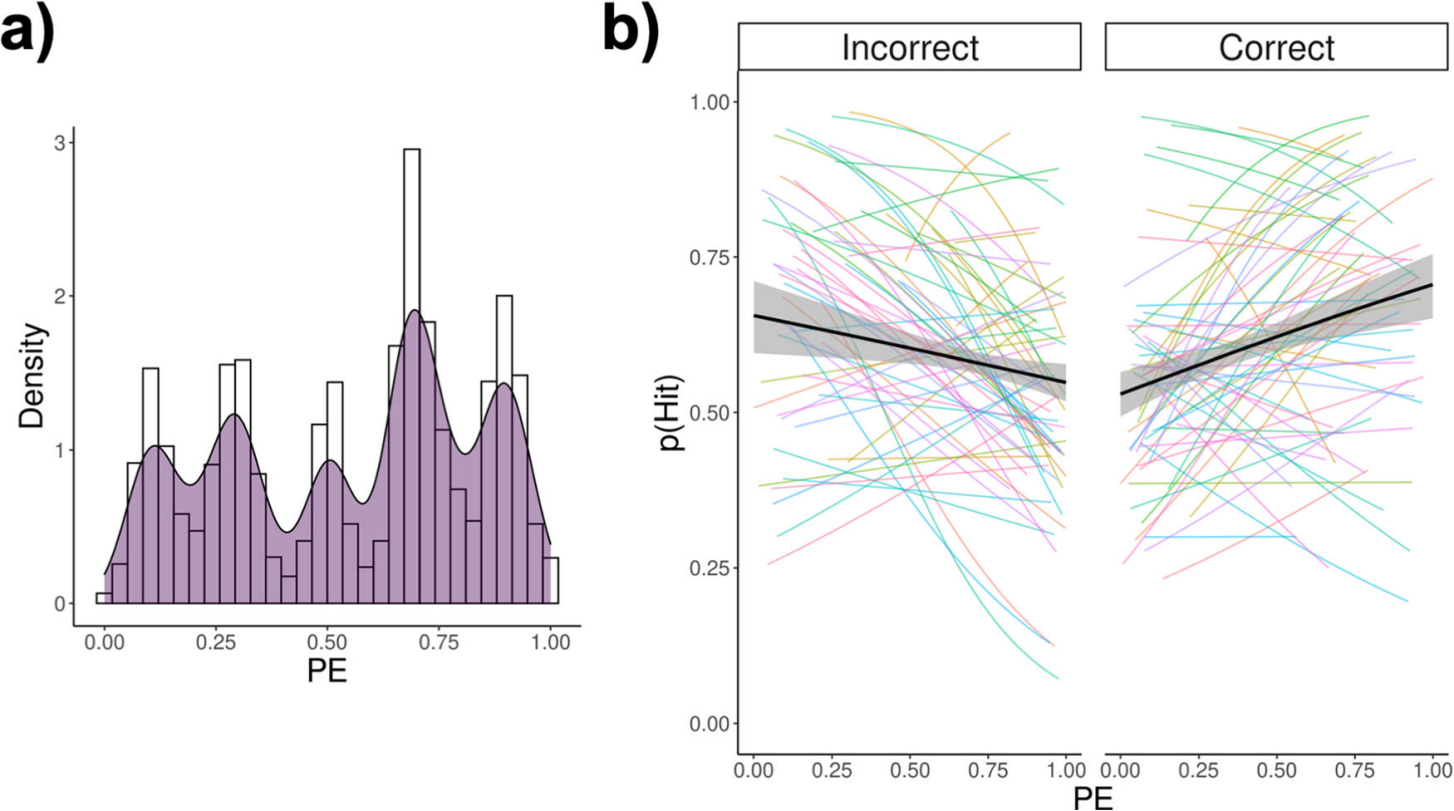
Computationally derived PE and memory. **a** Density plot and histogram of model-derived PE for merged data from Experiments 1 and 2. **b** Spaghetti plot of the observed relationship between PE and recognition memory as a function of prediction outcome. Each coloured line represents one participant, while the logistic regression line across participants is depicted in black.

As the two experiments were conceptually identical, the analyses were run on data collapsed across them (with the Experiment modelled as a factor). The distribution of PE by contingency collapsed across experiments is shown in Fig. 4a.

We then tested whether model-derived PE was related to recognition memory. We hypothesized either a positive relationship between PE and memory encoding, regardless of the outcome of participants’ predictions, or an interaction between PE and prediction outcome (see Fig. 1e). Plots of the observed values (see Fig. 4b) revealed a relationship between PE and memory modulated by prediction outcome. In order to statistically test for the significance of this relationship, we used a generalized linear-mixed model where participants were treated as random effects (see the “Methods” section). In the model, PE, learning rate, and prediction outcome, as well as their interactions, were added as fixed effects. In addition, random slopes for PE and prediction outcome, and their interaction, were also added to the model. Finally, the experiment was added as a fixed term (along with interactions) to model any differences between the two experiments. The results of the analysis are presented in Table 2.

**Table 2:** Results of the main analysis.

Fixed effects	*β* (se)	95% CI		*z*	*p*	OR
Intercept	0.66 (0.10)	0.46	0.85	26.54	*<*0.001	1.91
PE	−0.12 (0.19)	−0.45	0.22	−0.12	0.521	0.88
Prediction outcome^a^	0.10 (0.10)	−0.08	0.29	1.00	0.316	1.11
Learning rate	0.68 (2.30)	−3.74	5.19	0.30	0.767	1.98
Experiment^b^	−0.63 (0.20)	−1.00	−0.22	−3.15	0.002	0.53
PE × prediction outcome	1.87 (0.36)	1.18	2.53	5.11	*<*0.001	6.47
PE × learning rate	1.96 (3.67)	−3.26	6.83	0.53	0.593	7.11
Prediction outcome × learning rate	2.91 (2.13)	−0.50	6.34	1.37	0.171	18.40
PE × experiment	−0.09 (0.38)	−0.85	0.63	−0.25	0.801	0.91
Prediction outcome × experiment	0.19 (0.20)	−0.20	0.60	0.94	0.348	1.21
Learning rate × experiment	4.0 (4.58)	−4.83	13.15	0.87	0.381	55.03
PE × prediction outcome × learning rate	0.90 (5.84)	−5.75	6.80	0.15	0.877	2.47
PE × prediction outcome × experiment	−0.93 (0.74)	−2.28	0.47	−1.27	0.204	0.393
PE × learning rate × experiment	−1.95 (7.32)	−10.62	7.03	−0.27	0.790	0.141
Prediction outcome × learning rate × experiment	3.53 (4.23)	−3.00	10.39	0.83	0.404	34.23
PE × prediction outcome × learning rate × experiment	5.17 (10.96)	−2.09	12.93	0.47	0.637	175.88

Random effects	Variance	Std. dev.				
Subjects (Intercept)	0.30	0.55				
Prediction outcome	0.1	0.03				
PE	0.03	0.17				
Prediction outcome × PE	0.03	0.18				

^a^Prediction outcome contrasts have been set to 0.5 and −0.5 for correct and incorrect prediction, respectively.

^b^Experiment contrasts were set to 0.5 and −0.5 for Experiments 1 and 2, respectively.

Results showed that there was a significant interaction between PE and prediction outcome, ${\chi }_{(1)}^{2}$ = 26.14, *p* < 0.001, while the main effects of prediction outcome, learning rate, and the interaction between prediction outcome and learning rate, were not significant, *p*s > 0.316. There was also a significant main effect of the experiment, ${\chi }_{(1)}^{2}$ = 9.91, with participants performing worse overall at the recognition test in Experiment 2, compared to Experiment 1, *β* = −0.63, *p* = 0.002, OR = 0.53. Importantly, all the interactions including the experiment were non-significant, *p*s > 0.204, showing that the effects of interest did not differ across the two experiments. All the interactions including learning rate were also not significant, *p*s > 0.171, suggesting that the estimated learning rates did not affect overall memory accuracy and that also it did not modulate the effects of PE, prediction outcome, and memory. These results show that the effect of PE on memory encoding is different depending on the prediction outcome, supporting the hypothesis that choice outcome interacts with PE.

In order to break down the interaction between model-derived PE and prediction outcome, we analysed the effect of PE on recognition memory separately for correct and incorrect predictions. The analysis revealed a significant positive relationship between PE and recognition memory for correct prediction outcome, *β* = 0.80, *p* < 0.001, OR = 2.22, and a significant negative relationship between PE and prediction outcome for incorrect prediction outcome, *β* = −0.78, *p* < 0.001, OR = 0.46. These results show that PE improved recognition memory for correct predictions while impairing it for incorrect predictions.

We ran additional analyses with the hit rate binned by aggregating it between the quartiles for PE for each participant. Results from these analyses did not change the overall pattern presented in the previous analyses (see Supplemental Material and Fig. S8).

In the previous analyses, we considered the PE that was contingent on the category presented on each trial, independently of participants’ choice. However, PE can also be computed by incorporating participants’ prediction outcomes. Such PE is similar to the signed PE considered by previous studies^23,24^. The analysis of the effects of this kind of PE on recognition memory (included in Supplemental Material, see Fig. S9) showed a significant positive relationship between PE and recognition memory, with stronger positive PE related to better memory and stronger negative PE related to worse memory, which is in line with the results shown previously revealing an interaction between signed PE and prediction outcome.

## Discussion

Our brain extracts regularities from previous experiences and forms expectations accordingly in order to simplify the complexity of incoming information and better react to environmental demands. Events that mismatch expectations generate a PE that leads to the updating of expectations and is postulated to also affect the formation of episodic memories. Previous literature has provided mixed evidence on the effects of PE on memory formation, with studies on reward PE using reinforcement learning models producing contrasting results^21–24^. We explored the effects of PE on memory in a paradigm that did not include an explicit manipulation of the reward and conditions in which participants had already established prior expectations. In the task used, associations between context and object categories were first learned by participants and then used to predict the category of trial-unique upcoming objects. We used a reinforcement learning model to derive trial-by-trial PE generated by expectations of different strengths and analysed its effect on subsequent memory performance. We showed that the outcome of participants’ predictions was a modulator of the effects of PE on memory. Precisely, when a prediction turned out to be correct, higher PE was related to better memory; conversely, when a prediction turned out to be incorrect, lower PE was related to better memory. These results reveal a computationally specific effect of PE, highlighting the crucial modulating role of prediction outcome.

Even though our task did not involve any explicit reward, our findings are in line with studies on reward PE showing that positive prediction errors improve subsequent memory whereas negative prediction errors impair subsequent memory^23–25^, a pattern suggested to be related to dopaminergic activity promoting hippocampal plasticity and memory formation^28,29^. Thus, these results are in line with views suggesting that an intrinsic reward such as choice outcome might activate similar brain areas and neurotransmitters to the ones that are activated by a secondary reward (i.e., monetary reward^30,31^). It is well known in computational neuroscience that the neurotransmitter dopamine is responsible for a PE signal that drives plasticity in the striatum, facilitating repetitions of actions with better-than-expected outcomes^4,10^. Dopamine is also known to enhance long-term potentiation in the hippocampus^32^, and this modulatory effect might be responsible for the prioritization of relevant information in memory, such as rewarded stimuli^33,34^. The enhanced memory of rewarded information is thought to rely on the activity of hippocampal-enthorinal cortex microcircuitry, which has been shown to increase the representation of information as a function of the expected probability of a reward^35^.

It has also been shown that the effect of dopamine on the hippocampus can be bidirectional: Higher levels of dopamine cause phasic firing in the hippocampus which results in increased activation, while lower levels of dopamine produce tonic firing and inhibit hippocampal activation^29^. In the present study, such dopaminergic effects may have driven the difference between the expectations and the outcome of the prediction. More specifically, in conditions in which the expectation of a certain outcome was low, a correct prediction might nevertheless provide a PE signal which increases the release of dopamine in the striatum, promoting hippocampal activation, and resulting in better encoding. This idea is supported by evidence showing that increased striatum-hippocampus connectivity may lead to enhanced memory encoding^36^. Conversely, in conditions in which a certain outcome is strongly expected, an incorrect prediction might correspond to a negative PE, which suppresses the release of dopamine in the striatum and activation in the hippocampus, resulting in impaired encoding. Future studies investigating the connectivity between the hippocampus and striatum at these different conditions are needed to provide support for these hypotheses.

It should be noted that performance on the recognition task that we used may not be supported by the activation of the hippocampus. Recognition memory is thought to engage two separate processes: Recollection, known to rely on the hippocampus, and familiarity, known to be supported by extra-hippocampal structures^37^. The recognition memory task we used and the analysis we carried out does not allow us to conclude whether recognition performance is driven by recollection processes or by familiarity processes. Therefore, more studies are needed to investigate whether the pattern observed in the current study is supported by hippocampal or extra-hippocampal structures.

In the present study, PE was experienced at the time of the presentation of the to-be-remembered items. The objects presented provided feedback to participants on whether or not their predictions were correct. Previous studies from De Loof et al.^24^ and Jang et al.^23^ found effects of reward PE experienced during item presentation on memory encoding that are consistent with our results. Importantly, Jang and colleagues showed that memory effects of reward PE are elicited at item presentation, but not at the time of the presentation of the feedback when the objects were no longer shown. Therefore, our results provide additional support to the view that PE has to be elicited during object presentation in order to have solid effects on memory encoding.

Our results are partially in line with previous evidence showing a trade-off between predictions and episodic encoding. Sherman et al.^38^ showed that the act of predicting upcoming events based on learned regularities interfered with the encoding of new information. This activity was mediated by prediction-related activation of the hippocampus, suggesting that encoding is favoured in situations for which there are no strong predictive models. In the present findings, the improved memory for higher PE was observed in conditions in which the expectation for an outcome was low, and thus participants had not formed a clear predictive model yet. In addition, when participants incorrectly predicted the upcoming items, stronger expectations were also related to worse memory. It is also important to note that in the paradigm used in the current study predictions are generated at the categorical level, while memory is measured at the item level. The trade-off observed between predictions and episodic encoding may thus reflect the outcome of two competing mechanisms^38^.

Results from the current study are in contrast with previous findings showing a positive relationship between unsigned reward PE and memory^21,22^. In the present study, PE represented how unexpected the presentation of a category was and thus was equivalent to unsigned PE. In contrast to the findings from Rouhani and colleagues, our results showed that the overall effect of PE, independent of the outcome of the prediction, was not significant. One possible explanation for this discrepancy is the task that these studies used for encoding. Rouhani et al.^21,22^ presented participants with scenes that could be predictive of future rewards. After participants made their predictions, the images were presented together with the reward received, which could be either better or worse than expected, thus generating a reward PE. In their first study^21^, they showed a positive effect of unsigned prediction error on memory encoding, which thus improved memory encoding for both better- and worse-than-expected outcomes. However, it was not clear whether the effect was due to PE occurring before or after the feedback presentation, as the images were presented even before the presentation of the feedback. In a second study^22^, the authors manipulated reward PE before and during feedback delivery separately, finding an effect of signed reward PE for images presented before feedback delivery and an effect of unsigned reward PE for items presented during feedback presentation. The discrepancy between these findings and our findings could be due to the different methodologies used to elicit PE. The reward PE experienced during feedback delivery in the study by Rohuani and colleagues^22^ was driven by a specific condition in which participants could win or lose money. As a consequence, the effects observed might have been triggered by arousal-related prediction error, which has been shown to have emotional processes linked to the activity of the amygdala^39^, which are in turn known to enhance memory^40^. Evidence showing that arousal-related unsigned prediction error is linked to enhanced memory encoding is in line with this explanation^41^.

It is important to note that mismatched information might have been discarded as not helpful for the future because the task used in both experiments included contingencies that were established before the encoding of the events and never changed during the course of the tasks. Participants underwent extensive learning phases in which they established their expectations for the different contexts prior to the encoding task. This setting has not frequently been used in previous computational modelling studies, although it is more common in real, daily life that people find themselves in situations they are familiar with. In conditions in which expectations are established and known to be stable, deviant information may be taken as a rare event of chance. On the contrary, it is possible that mismatched information would be more valued during the learning phases or in conditions where changes are expected to occur. Evidence showing different behavioural and neurophysiological correlates of expected and unexpected uncertainty is in line with this view^42^. For example, in tasks in which the contingencies are stable, encountering low-probability trials is expected, and the value of those stimuli in predicting future events is suppressed via cholinergic neurotransmission^43^. In contrast, in tasks in which the probabilistic structure of the environment changes unexpectedly, encountering low-probability stimuli boosts learning through the release of norepinephrine^42^, an effect that might also result in increased episodic memory encoding.

To characterize the contingency-learning process we fitted four different reinforcement learning models to participants’ data: An optimal model with a decreasing learning rate that was the same for each participant, a model with a decreasing learning rate that was free to vary across participants, a free-learning rate model considering the outcome of participants’ choice (evaluative model), and a free-learning rate outcome-free model considering the information given on each trial (instructive model). Model comparison showed that participants’ learning processes did not conform to the normative behaviour of a Bayesian model, which prescribes that the optimal way of learning in this task entails decreasing the learning rate over trials. In fact, participants’ data were best explained by models estimating individual, constant learning rates. In addition, in the best-fitting model the context-category associations were learned by increasing the strength of the associations of the category presented on a given trial and decreasing the strength of the associations of the categories not presented, regardless of participants’ choice and its outcome. This result suggests that prediction outcome might not be important for PE-driven incremental learning of associations, while it is crucial for modulating the influence of PE on the encoding of item identity.

Our results also showed that the estimated learning rate did not affect overall recognition memory. This can potentially be explained by the fact that the estimated learning rate was fixed throughout the experiment. In our study, a learning rate was estimated for each individual, akin to an individual difference measure. It is possible that the potential effect of learning rate on memory is rather a within-person process, observable only in paradigms in which learning rate changes (for example, when environmental contingencies change^42^). It is also important to note that our interest focused on conditions where the contingencies were already learned, while its effects during the learning of the contingencies were not addressed. However, the effects of the learning rate might vary considerably in conditions in which participants learn the contingencies. Therefore, future studies are needed to examine the dynamic relationships between learning rate and memory over time.

In conclusion, the current study provides evidence of the effects of PE on memory encoding. In conditions where no explicit reward is delivered, we show that the effects of computationally derived PE on memory are modulated by whether or not a prediction is correct, hence informing future studies exploring the interactions between learning and memory.

## Methods

### Participants

As Experiments 1 and 2 were conceptual replications with a similar design, both experiments are described together in the following. Differences between the experiments are pointed out.

In Experiment 1, 32 young adults (20 female; mean age = 22.59 years, s.d. = 3.18) were recruited through advertisements placed at the Goethe University campi in Frankfurt am Main. In exchange for participation, participants received either course credits or a monetary reimbursement of 8€/h. In Experiment 2, 40 participants (19 female; mean age = 24.87, s.d. = 4.64) were recruited through the Prolific platform (https://www.prolific.co/). All participants had normal or corrected-to-normal vision and no history of psychological or neurological disorders. All participants gave written informed consent prior to participation. In exchange for participation, volunteers received either course credits or reimbursement of 8€/h. The study was approved by the ethics committee of the Goethe University Frankfurt am Main.

### Materials

For a more detailed description of the materials and methods used, please refer to the original publication^26^. For Experiment 1, six coloured scene categories depicting real-world outdoor locations were taken from the ECOS database (https://sites.google.com/view/ecosdatabase/) were used as contexts (see Fig. 1). The selected scene categories were beach, mountain, road, desert, savannah, and seabed. As objects, 192 coloured images depicting real-world objects were collected from an online search and were used as target objects. The images selected included the same number of objects for three different object categories: musical instruments, fruits/vegetables, and household objects. All images were subjected to creative commons licensing and are available at https://github.com/ortiztud/premup. For Experiment 2, the number and types of scene categories were the same as in Experiment 1. However, the object categories were reduced and only two were used: musical instruments and household objects.

### Design and procedure

In Experiment 1, participants completed the learning, encoding, and retrieval phases in one session, while in Experiment 2 participants completed the learning phase in the first session and the encoding and retrieval phase ~24 h later. In addition, in the second session of Experiment 2 participants worked on an extra reminder block of contingency learning before the encoding phase. In Experiment 1, stimulus presentation and recording of the responses were done using Matlab’s Psychtoolbox^44^ and a 60 Hz monitor (resolution: 1680 × 1050, full HD). Experiment 2 was moved online due to the COVID-19 pandemic, and some necessary changes were implemented. Stimulus presentation and response collection were programmed in PsychoPy (v2021.1.4) and hosted online on Pavlovia (https://pavlovia.org). At the beginning of each session, the experimenter met the participant in a virtual room using an online video-conferencing tool, during which the appropriateness of the testing setup was assessed with a brief set of questions about the participant’s overall well-being, about the physical room in which the task would be performed and about the computer that would be used. Experimenters ensured that all participants were sitting in a quiet room, used a laptop or a desktop computer, and were encouraged to minimize distractions as much as possible during the session. At the end of the session, the experimenter met the participant again and asked them about any unforeseen event or situation that might have come up during the completion of the task. Finally, to maximize engagement, self-administered breaks were included after every 40 trials during the contingency learning and encoding phases.

#### Contingency learning phase

Participants were presented with the scene contexts and were instructed to learn which object category was more likely to belong to each of the scene contexts; they were told that some contexts were easier to learn than others, but the exact contingencies were not explicitly given. A fixation cross at the centre of the screen marked the beginning of each trial and lasted for 500 ms. After that, a scene image including a rectangular white patch with a question mark was presented. They were then asked to make a prediction about the object category that they thought they would encounter in that context. Three response alternatives were given for Experiment 1 (i.e., musical instruments, fruits/vegetables, and household objects) and two for Experiment 2 (i.e., musical instruments and household objects). Category reminders were placed at the bottom of the screen and participants could choose among them by pressing one of three arrow keys in Experiment 1 (left arrow, down arrow, right arrow) and two arrow keys in Experiment 2 (left arrow, right arrow). The selected category was highlighted with a yellow frame. After 2 s from the scene onset, the question mark within the white patch was replaced by an object and the coloured frame changed colour to indicate a correct or incorrect response. Specifically, the red frame indicated incorrect responses, green frame indicated correct responses. Objects and feedback were shown on the screen for 1 s. Participants were told to use the feedback for learning the contingencies over trials.

The frequency to which an object category was encountered in the given scene contexts was manipulated to create different expectations. In Experiment 1, three object categories were used for each scene context. In half of the scene contexts, one of the three object categories was frequently presented 80% of the trials, while the other two were equally presented in 10% of the trials each (0.80–0.20 contingencies). Conversely, in the other half of the contexts, the object categories were all three equally probable, being presented each 33% of trials (0.33–0.33–0.33 contingencies). In Experiment 2, in two context scenes, one of the two object categories was presented in 90% of the trials, while the other object category was presented in 10% of the trials (0.90–0.10 contingencies). In two more scene contexts, the more frequently presented object category was shown in 70% of the trials, while the other object category appeared in 30% of the trials (0.70–0.30 contingencies). Finally, in two scene contexts, both object categories were equally likely to be presented, appearing each one in 50% of the trials (0.50–0.50 contingencies). To achieve the desired contingencies without proportionally increasing the number of individual objects used, different objects were repeated a different number of times depending on their category and the contexts in which they were shown. The association of each object category to each scene category was counterbalanced across participants so that across the entire sample, every object category was paired with every scene category.

#### Encoding phase

The encoding Phase in Experiments 1 and 2 was similar to the learning phase, with only minor changes introduced. The explicit feedback represented by the coloured squared surrounding the object was removed in this phase. We included this additional explicit feedback in the learning phase to help participants familiarize themselves with the task and to speed up the contingency learning process. However, as the critical phase for testing the PE-related effects on memory was the encoding phase, we removed this colour information to avoid potential contamination of arousal and valence on memory (see, e.g., ref. ^45^). In addition, a new set of objects was used, and each of these objects was presented only once. In Experiment 1, we had 24 objects for each of the three 0.33–0.33–0.33 contexts, and 24 objects for each of the three 0.80–0.20 scene contexts, for a total of 144 objects. In the 0.80–0.20 contexts, 24 filler objects were added for the object categories presented 80% of the time. Each of these 24 objects was repeated seven times to reach the desired contingency. Therefore, the total number of trials in the encoding phase of Experiment 1 was 312: 240 for the 0.80–0.20 condition, and 72 for the 0.33 condition. In Experiment 2, 20 objects for each of the two 0.90–0.10 and 0.70–0.30 contexts were presented only once. Then, to achieve the desired contingencies for each scene category, we used five filler objects for the 0.90 and five filler objects for the 0.70 contingency category. These filler objects were repeated 16 times for the 0.90 contingency category and three times for the 0.70 contingency category. For each of the two 0.50–0.50 contexts, we used 10 objects, which were presented only once, and 10 fillers, each repeated twice. In total, the number of trials in the encoding phase of Experiment 2 was 330: 200 in the 0.90–010 condition, 70 in the 0.70–0.30 condition, and 60 in the 0.50 condition. Filler trials from both experiments were not considered in the analysis of recognition memory (see Table S1 in the Supplemental Material for a breakdown of the number of objects for each context and phase).

Similarly to the contingency learning phase, the participant’s task was to predict which object category followed a scene context that was presented on every trial. The contingencies between object categories and scenes were the same as in the previous learning phase.

#### Retrieval phase

In the object recognition test, all the objects from the encoding phase together with new objects were used. In Experiment 1, the hit rate was calculated based on a sample of half of the 144 objects (72 trials), as half of the trials were selected for the immediate recognition session which is the focus of the analysis of the current study. The rest of the trials were selected for a delayed recognition test, which was added to explore the potential modulating factor of consolidation on the interplay between PE and memory and it is not considered in the current study. In addition to the 144 old trials, 144 new items were presented in the recognition memory task. In Experiment 2, all the 100 old objects were tested in the immediate recognition session and thus included in the hit rate calculation, together with 80 new items. The new items belonged to the same categories as the items presented during the encoding phase, so that in Experiment 1 one-third of the new items were musical instruments, one-third were fruits/vegetables, and one-third of household objects; in Experiment 2 half of the items were musical instruments and half household objects (see Table S1 in the Supplemental Material for the exact number of objects for each category and phase). Trials started with a fixation cross for 500 ms, and objects were presented in isolation at the centre of the screen. Participants were required to make old/new judgements. All the responses in the retrieval phase were self-paced and not time-constrained, and the display stayed unaltered until participants made a response. After that, a new trial was presented.

To evaluate overall memory performance for each participant, a d’ score was calculated from the hits (responding “old” to old items) and false alarms (responding “old” to new items), an index that indicates participants’ ability to discriminate between old and new items. In order to exclude participants who did not perform the task above the chance level, we created a null distribution by generating 5000 random permutations of the trial labels. We then excluded participants whose performance was below the 95% percentile of the null distribution. Five participants from Experiment 1 and five from Experiment 2 with an overall *d*’ score below the obtained threshold were excluded from further analyses. After the exclusion, the final *d*’ was *d*’ = 0.93, *t*(26) = 13.7, *p* < 0.001 for Experiment 1, and *d*’ = 0.90, *t*(34) = 16.8, *p* < 0.001 for Experiment 2, indicating that participants were overall able to discriminate previously presented old items from new distractors.

### Computational models

We fitted participants’ contingency learning and encoding data with computational models. The models considered are all different versions of a standard Rescorla-Wagner model (or *Q*-learning)^3,7^. For each scene category, the model estimates a trial-level variable *Q* for each object category included in the experiments (three in Experiment 1 and two in Experiment 2). These *Q* values reflect the strength of participants’ belief that a certain object category (for example, “Musical instruments”) will be presented in a specific context (for example, “Beach”). Since we have *N* object categories for each *n* context, the estimates *Q* of the probabilities can be represented by the following *j*-by-*c* matrix:

1 $$\left[\begin{array}{ccc}{Q}^{1,1}, & {Q}^{1,2}, & \ldots\\ {Q}^{2,1}, & {Q}^{2,2}, & \ldots \\ \ldots, & \ldots, & {Q}^{j,c}\end{array}\right]$$

where *Q*^1,1^ represent the expected value *Q* for category *j* = 1 in context *c* = 1. For all the models considered in this study, the estimated values are stored in a category *j* by context *c* matrix as this one and initialized as *Q*^*j,c*^ = 0.33 in Experiment 1, and *Q*^*j,c*^ = 0.5 in Experiment 2.

#### Decreasing learning rate instructive model (dLRI)

First, to provide a normative Bayesian solution, we used a Dirichlet-multinomial model that we re-formulated to a delta-rule model (see Supplemental Material). This model learned according to prediction errors scaled by a learning rate to produce an update of the estimated category probabilities. The learning rate dynamically decreased across trials so that the model learned more rapidly at the beginning of the task, and more slowly on later trials. Formally, the model sequentially updated the category probabilities for each context according to

2 $${Q}_{t+1}^{j,c}={Q}_{t}^{j,c}+\frac{1}{t}{\delta }_{t}^{j,c},$$

where ${Q}_{t+1}^{j,c}$ denotes the estimate of the probability of category *j* in context *c* at the next trial *t* + 1. This estimate is based on the current estimate of the category probabilities ${Q}_{t}^{j,c}$ and the prediction error ${\delta }_{t}^{j,c}$, calculated as

3 $${{\delta }_{t}}^{j,c}={r}_{t}^{j}-{Q}_{t}^{j,c},$$

where the feedback ${r}_{t}^{j}$ represents an array of *N-by-j* elements, in which each element refers to a category *j*, and it is defined as follows:

4 $${r}_{t}^{j}=\left\{\begin{array}{l}1\,{\rm {if}}\,j={j}_{t}\\ 0\,{\rm {otherwise}}\end{array}.\right.$$

The values of the array are 1 if category *j* is present on trial *t*, and 0 if it is not. Therefore the model is assuming that a value estimate for an object category that appears on a trial incrementally increases as a result of a prediction error until ${Q}_{t}^{j,c}$ reaches its asymptote of 1. Conversely, the value estimates of categories that are not presented on trial *t* decrease as a result of a negative prediction error, unless ${Q}_{t}^{j,c}$ for those categories has already a value of 0. Therefore, this model only uses instructive feedback, which indicates what is the correct choice, independently of participants’ actions. The learning rate $1/t=:\alpha$ indicates to which degree the prediction error influences the updated estimate of the category probabilities. Given that the learning rate in our case directly depends on the number of completed trials *t* for a context *c*, it continuously decays as a function of trials. This principle ensures that the influence of prediction errors is stronger at the beginning of the task.

#### Decreasing free learning rate instructive model (dfLRI)

The dLRI shows how an optimal agent should update the expected values. However, participants’ behaviour may be far from optimal. For this reason, the dfLRI allows each participant to have its own learning rate *α*, which decreases as a function of the trial number, similarly as in the dLRI model:

5 $${Q}_{t+1}^{j,c}={Q}_{t}^{j,c}+\alpha \frac{1}{t}{\delta }_{t}^{j,c},$$

where ${Q}_{t+1}^{j,c}$ and ${\delta }_{t}^{j,c}$ are estimated as in the previous model (dLRI).

#### Free learning rate instructive model (fLRI)

This model estimates the learning rate by the participant, as in the previous dfLRI model. However, the present model considers the learning rate *α* to remain constant throughout the learning and encoding phases. The expected values are thus updated according to the following rule:

6 $${Q}_{t+1}^{j,c}={Q}_{t}^{j,c}+\alpha {\delta }_{t}^{j,c},$$

while ${\delta }_{t}^{j,c}$ is the same as in Eq. (3). Also, note that this model uses the same instructive feedback as in Eq. (4).

#### Free learning rate evaluative model (fLRE)

This model still allows participants to have a fixed learning rate *α*. However, this model assumes that the feedback depends on the actions that participants take. The expected values are thus updated as follows:

7 $${Q}_{t+1}^{j,c}=\left\{\begin{array}{l}{Q}_{t}^{j,c}+\alpha {\delta }_{t}^{j,c}\,{\rm {if}}\,{a}_{t}={j}_{t}\\ {Q}_{t}\,{\rm {otherwise}}\end{array}.\right.$$

where *a*_*t*_ is the object category selected by participants on a given trial, ${\delta }_{t}^{j,c}$ is calculated as in Eq. (3), and *r*_*t*_ is 1 if the choice is the correct one, and 0 otherwise.

#### Action selection

The expected *Q*-values computed through the models listed above were translated into choice probabilities by implementing a softmax rule as follows:

8 $${P}_{t}^{j,c}=\frac{\exp (\beta {Q}_{t}^{j,c})}{{\sum }_{n=1}^{j}\exp (\beta {Q}_{t}^{c})},$$

where ${P}_{t}^{j,c}$ represents the probability of choosing a specific object-category *j* for a defined scene category *c*. The inverse temperature parameter *β* is another free parameter that modulates the stochasticity of the choice, with higher values meaning more deterministic actions and lower values more stochastic choices.

#### Parameter recovery

Before fitting the models to participants’ data, a parameter recovery procedure was run for both Experiments 1 and 2, in order to check whether the fitting procedure for each model gave meaningful parameters and to find potential parameter boundaries. *Surrogate* data with randomly sampled known parameters were simulated, and then the models were fit to the simulated data (see ref. ^46^). The priors from which the simulation parameters were sampled are shown in Table 3. In order to fit the data, we used maximum likelihood estimation (see subsection “Parameter estimation and model comparison”). Because the models are designed to reflect participants’ learning, only the 0.80–0.20 (Experiment 1) and 0.90–0.10 and 0.70–0.30 (Experiment 2) conditions were simulated. A high correlation between simulated and fitted data indicates that the model successfully recovered the parameters that were used to generate the data. First attempts to recover the parameters allowed to set the boundaries for the inverse temperature parameters. Plots of the parameter recovery are shown in Figs. S2 and S3.

**Table 3:** Priors for the parameters.

Parameter	Priors
*α*	~*U*(0,1)
*β*	~exp(1)

#### Model recovery

Besides parameter recovery, another procedure to evaluate the reliability of a model is model recovery^46^. The aim of model recovery is to determine that a model, among the models of the model space, can successfully be identified as the generative model. To achieve this, data from the four different models were simulated (with randomly sampled parameters) and then fit each of the models. The models were then compared to determine which one fitted the data best. The method used to assess the fit of the models was the Bayesian information criterion (BIC) which incorporates a penalty for the number of parameters:

9 $${\rm {BIC}}=-2{\rm {log}}\widehat{{{LL}}}+{k}_{m}{\rm {log}}(T),$$

where $\widehat{LL}$ is the log-likelihood value when the model is fitted with the best fitting parameter, and *K*_*m*_ is the number of parameters in the model *m*. Lower values of BIC mean better fit. The comparison between the models for each set of generated data was repeated 100 times to generate the confusion matrices shown in Fig. S4.

### Parameter estimation and model comparison

The models were finally fit to participants’ data to estimate the parameters. The parameters of best fit for each model were estimated through maximum likelihood estimation. This procedure allowed us to find the parameters *θ* that maximize the likelihood of the data given the parameters $p({d}_{1:t}|\theta ,m)$. The probability of the whole dataset *d* is calculated as the product of the choice probabilities $p({c}_{t}|{d}_{1:t-1},\theta ,m)$. As the product of the choice probabilities is often a very small number, it is common practice to use the log-likelihood instead, which is the sum of the log of the choice probabilities^7,46^:

10 $${{LL}}=\mathop{\sum }\limits_{t=1}^{n}{\rm {log}}\,p({c}_{t}|{d}_{1:t-1},\theta ,m)$$

The search over the full set of free parameters was optimized through the package *optim* in R, which was fed with the negative log-likelihood and a set of starting points randomly selected from the priors shown in Table 3. The *α* parameter was constrained between 0 and 1, while the *β* parameter between 0 and 10, as parameter recovery showed that for values that exceeded 10, the model could not distinguish between different beta parameters. Because the optimizer may find a local rather than a global minimum, we ran the search for the best parameters five times, starting from different points, and then used the best-fitting parameters among the five iterations, i.e., the parameters that minimized the log-likelihood. After estimating the parameters, a *BIC* value (see Eq. (9)) was computed for each model and for each participant using the parameters of best fit.

To compare the fit of the models, we calculated the average BIC across all subjects for each model, then counted the number of participants for which each model was the best fit. In addition, we used the model evidence of the best model within each participant: Following^47^ and^48^, model evidence was defined as “weak”, “positive”, “strong”, or “very strong” depending on the BIC difference between the best and the second best model for each participant. Precisely, evidence was “weak” when the BIC difference between the best and the second best model was below 2, “positive” when it was between 2 and 6, “strong” when it was between 6 and 10, and “very strong” when it was above 10.

### Statistical analysis

In order to test the statistical significance of the effects of interest, we used linear mixed-effect models and generalized linear mixed-effect models, implemented in R^49^ through the package *lme4*^50^. Because our main outcome variable (memory) is binary, we used the logit link function in the binomial family to fit the models to accuracy data. Participants were modelled as random intercepts, while the explanatory variables and their interactions were modelled as both fixed and random effects. The generalized linear mixed-effect model for the analysis of the effects of the computationally derived PE on memory can be formalized as the following:

11 $$p{({\rm {hit}})}_{i,j}=\frac{1}{1+{\rm {exp}}-({\beta }_{0,j}+{\beta }_{{1}_{j}}{\rm {PE}}+{\beta }_{{2}_{j}}{\rm {PO}}+{\beta }_{{3}_{j}}{\rm {PE}}\cdot {\rm {PO}}+{{\epsilon }}_{i,j})}.$$

The formula represents the probability of correctly recognizing an object *i* for a participant *j*. The intercept *β*_0,*j*_ is composed of a common (fixed) intercept for the population (i.e., the average *p*(hit)) plus a subject-specific random effect. *β*_1,*j*_ PE and *β*_2,*j*_ PO represent the slopes for PE and prediction outcome, respectively, while *β*_3,*j*_PE PO refers to their interaction. Each of the slope coefficients is formed by a common (fixed) slope for the population level plus a subject-specific random slope. Finally, *∈*_*i,j*_ represents the within-participant residual term. Note that this formula does not include the effect of the experiment, which varied between participants, and thus were included in the generalized mixed-effect model only as fixed effects and fixed interaction terms. The variance–covariance matrix for the random effects was set as unstructured so that the covariances between the random terms could take any finite positive value. Therefore, we used the maximal random effect structure justified by the design^51^. To test the significance of the parameters, a Wald chi-square test was used. Effect sizes were reported as odds ratio (exp(*β*)), which represents the change in odds. The odds are in turn calculated as follows:

12 $${\rm {Odds}}=\frac{p({\rm {hit}}=1)}{1-p({\rm {hit}}=1)}.$$

For the analysis of recognition memory as a function of contingency condition and prediction outcome, and for the analysis of the effect of binned PE, we used linear mixed-effect models with hit rate as response variable:

13 $${\rm{Hit}}\,{\rm{Rate}}=\frac{{\rm {hits}}}{{\rm{hits}}\,{+}\,{\rm{missed}}}.$$

Binned PE was treated as a categorical variable with four levels. Testing for the significance of planned contrasts was corrected for multiple comparisons by using Bonferroni correction:

14 $${p}_{{\rm {corr}}}=p\cdot k,$$

where *p* is the *p*-value of the comparison and *k* is the overall number of comparisons considered in a model. All tests were two-tailed.

### Reporting summary

Further information on research design is available in the Nature Research Reporting Summary linked to this article.

## Supplementary information

Supplementary material

reporting summary

## Data Availability

All behavioural data that support the findings of this study have been made available on the Open Science Framework (https://osf.io/97pu8/) with identifier 10.17605/OSF.IO/97PU8.

## Code availability

All model codes and custom analysis scripts that support the findings of this study have been made available on the Open Science Framework (https://osf.io/97pu8/) with the identifier 10.17605/OSF.IO/97PU8.
