# Supplementary material for: The effect of prediction error on episodic memory encoding is modulated by the outcome of the predictions
Source: NPJ Sci Learn. 2023 May 29;8:18. doi: 10.1038/s41539-023-00166-x (PMC10226026; doi:10.1038/s41539-023-00166-x)
Supplement: Supplementary file 1 — Supplementary material [file 41539_2023_166_MOESM1_ESM.pdf]

Supplemental Materials

Memory Performance

We analyzed recognition memory for both Experiment 1 and 2 as a function of prediction outcome and of the contingency condition of the category predicted by participants (Figure S1). Contingency condition of the predicted category represents the contingency condition related to the object category predicted by participants on a given trial. For example, the condition “0.80” includes situations in which participants are predicting an object category presented 80 % of the times on a given scene (e.g., with reference to Figure 1, predicting "Musical Instrument" when cued with a "mountains" scene). By contrast, the condition “0.20” includes situations in which participants are predicting an object category presented 20 % of the times on a given scene (e.g., with reference to Figure 1, predicting either "household objects" or "fruits/vegetables" when cued with a "mountains" scene).

In Experiment 1, there was a significant interaction between prediction outcome and contingency condition, $\chi^2_{(1)} = 9.40$, $p = .009$. To break down the interaction, recognition memory was analyzed separately for incorrect and correct predictions. For correct predictions, the difference between the contingency conditions 0.33 and 0.80 was significant, $\beta = 0.86$, p_{corr} <.001, OR = 2.36, showing that the items presented when participants correctly predicted the 0.33 category were remembered better than the items presented when participants correctly predicted the 0.80 category. In addition, there was a trend for improved memory for items presented when correctly choosing the 0.33 compared to the items presented when participants correctly predicted the 0.20 category, $\beta = 01.09$, p_{corr} =.056, OR = 2.99. The other pairwise comparison did not reach significance, $p_{corr} = 1$. For incorrect predictions, memory tended to be worse for higher predicted contingency conditions, but this did not reach significance, $\chi^2_{(1)} = 4.00$, $p = .133$. In Experiment 2, there was a main effect of contingency condition, $\chi^2_{(2)} = 13.56$, $p = .009$, while the main effect of prediction accuracy and the interaction between prediction outcome and predicted contingency were not significant, $p\text{s}_{corr} > .113$. Pairwise comparisons showed that predicting the contingency condition 0.50 led to improved recognition for the items, compared to predicting the contingency condition 0.90, $\beta = 0.34$, p_{corr} =.010, OR = 1.41. Predicting the contingency condition 0.70 also led to improved item memory, compared to predicting the contingency condition 0.90, $\beta = 0.27$, p_{corr} =.007, OR = 1.31. The other comparisons did not reach significance, $p\text{s}_{corr} > .170$.

Taken together, these results show that the contingency condition of the category predicted affected the likelihood of remembering the object presented, and that this effect was partially modulated by whether the prediction was correct or incorrect. Specifically, for

incorrect predictions, predicting an object category which belonged to a higher contingency condition tended to be detrimental to memory. Incorrectly predicting higher contingency condition category is a condition that is likely to generate higher aggregated PE, because participants' expectations were higher and thus they tended to predict the most likely category. For correct predictions, predicting categories belonging to lower contingency conditions (0.5 contingency condition in Experiment 1, 0.5 and 0.7 contingency conditions in Experiment 2) tended to lead to better memory. These conditions are likely to generate higher aggregated PE, as participants' expectations were lower.

Dirichlet-Multinomial Model

We used the Dirichlet-multinomial model to formalize learning as optimal Bayesian updating of the Dirichlet distribution (1). We apply the Multinomial distribution because the Categorical distribution that we use in our task is a special case of the Multinomial distribution where only one outcome is sampled on each trial. That is, the outcomes x of the task were drawn from a Multinomial distribution

$$\mathrm{Mu}(\mathbf{x}|1, \boldsymbol{\theta}) =: \mathrm{Cat}(x|\boldsymbol{\theta}) \tag{S1}$$

where $p(x = j|\boldsymbol{\theta}) = \theta_j$. For example, in the weak prior condition, $\boldsymbol{\theta} = [0.33,\ 0.33,\ 0.33]$. The Dirichlet distribution is the conjugate distribution of the Multinomial distribution and can therefore be utilized as a prior. This distribution is parameterized by the concentration parameters $\boldsymbol{\alpha} = (\alpha_1, \alpha_2, ..., \alpha_J)$.

We use the Dirichlet distribution to model the participants' prior expectations (i.e., at the beginning of the learning phase) about the category probabilities, which are often called pseudo-counts. Here we assume that participants start the task with a flat prior that reflects that all categories are equally likely, which corresponds to $\boldsymbol{\alpha} = (1, 1, ..., 1)$. These values thus indicate the assumption that each category has been pseudo-counted once.

To obtain the posterior, the only operation required is adding the observed data to the prior. In order to obtain an estimate of the category probabilities, we can compute the expected value of the posterior, referred to as the maximum a posteriori (MAP) estimate:

$$\hat{\theta}_j = \frac{T_j + \alpha_j - 1}{T + \alpha_0 - J}. \tag{S2}$$

T_j denotes how many times category j was presented and T refers to the total number of trials. Under the assumption that $\boldsymbol{\alpha} = (1, 1, ..., 1)$, the MAP estimate is equal to the maximum likelihood (ML) estimate that is based on the empirically observed frequency

of the categories:

$$\hat{\theta}_j = \frac{T_j}{T}. \tag{S3}$$

Delta-Rule Formulation

We now show how eq. (S3) can be translated into the delta rule. Let $\hat{\theta}_{t,j}$ denote the estimate of the jth category probability on trial t. Then the estimate of the jth category on trial $t+1$, denoted by $\hat{\theta}_{t+1,j}$, can be computed according to

$$\begin{aligned}
\hat{\theta}_{t+1,j} &= \frac{t_j}{t} \\
&= \frac{1}{t} t_j \\
&= \frac{1}{t} \sum_{i=1}^{t} x_{i,j} \\
&= \frac{1}{t} \Big(x_{t,j} + \sum_{i=1}^{t-1} x_{i,j} \Big) \\
&= \frac{1}{t} \Big(x_{t,j} + (t-1) \frac{1}{t-1} \sum_{i=1}^{t-1} x_{i,j} \Big) \\
&= \frac{1}{t} \Big(x_{t,j} + (t-1)\hat{\theta}_{t,j} \Big) \\
&= \frac{1}{t} \Big(x_{t,j} + t\hat{\theta}_{t,j} - \hat{\theta}_{t,j} \Big) \\
&= \hat{\theta}_{t,j} + \frac{1}{t}(x_{t,j} - \hat{\theta}_{t,j}) \\
&= \hat{\theta}_{t,j} + \alpha_t \delta_{t,j},
\end{aligned} \tag{S4}$$

where $\delta_{t,j} := (x_{t,j} - \hat{\theta}_{t,j})$ corresponds to the prediction error and $\alpha_t := \frac{1}{t}$ is the learning rate (2).

Parameter Recovery

In order to check whether the models could successfully recover the parameters, *fake* data were first simulated with known parameters. Next, models were fit to the simulated data and parameters of best fit were estimated. Finally, the recovered parameters were compared with the known parameters. Graphs showing the results of parameter recovery are presented in Figure S2 and S3. High correlation between the simulated and fitted parameters indicates successful recovery. Note that the inverse temperature parameter was constrained between 0 and 10 as previous parameter recovery attempts showed that recovery was not reliable for parameters above that range.

Model Recovery

To assess the ability of a model to successfully distinguish between different models, a model recovery procedure was used. Data from the three different models were simulated and then

fit to each of the models to determine which model fit best. This procedure was repeated 100 times. The confusion matrices shown in Figure S4 show the results of this procedure. Each cell represents the probability of data simulated by models in the X axis to be best fit by models in the Y axis. Higher probabilities in the diagonal means that the models can successfully recover the models from which the data were generated.

Simulated and Empirical Comparison for dLRI, dfLRI, and fLRE Models

Figure S5 shows comparisons between simulated and actual data for the instructive model with a decreasing learning rate (dLRI), the instructive model with a decreasing learning rate that was free to vary between participants (dfLRI), and the evaluative model with a free learning rate (fLRE).

Learning Rate Validation

To check whether the model captured participants' differences in learning rate, we compared cumulative accuracy for simulated and empirical data at different values of learning rate α. For simulated and empirical data, quartiles for α were calculated (zeroth, first, second, third, and fourth quartile), and cumulative accuracy was aggregated for the data points between one quartile and the previous one. Cumulative accuracy for the four bins created is shown in Figure S6, as a function of order of type of data (empirical vs simulated), and Experiment (1 vs 2). In both Experiments, the simulated data mirrored the pattern of the actual data, showing that the model was capable of capturing observed effects of individual differences in learning rate on cumulative accuracy.

Analysis with Binned Hit Rate

In order to compare memory at different levels of the model-derived PE, we calculated the quartiles for PE for each participant, separately for trials with correct and incorrect prediction outcome. We binned the hit rate by aggregating it between the quartiles, to create four bins which eventually were used as the explanatory variable in our analysis. A graph with the distribution of PE by binned data is shown in Figure S7.

Hit rate as a function of binned PE and prediction outcome is shown in Figure S8. We then tested for the three-way interaction between binned PE, prediction outcome, and experiment, in a linear mixed-effects model, adding participants as random effects. The three-way interaction was not significant, $\chi^2_{(3)} = 4.23$, $p = .238$. In addition, the interactions between PE and experiment, and the interaction between prediction outcome and exper-

iment were not significant ($\chi^2_{(3)}$ = 1.68, p = .642, $\chi^2_{(3)}$ = 0.81, p = 0.368, respectively). These results suggest that there were no significant differences in the effects of PE and in the interaction between PE and prediction outcome between the two experiments. By contrast, there was a main effect of Experiment, $\chi^2_{(3)}$ = 7.70, p = .005, showing that overall participants' performance was significantly worse in Experiment 2, compared to Experiment 1. Importantly, there was also a significant interaction between PE and prediction outcome, $\chi^2_{(3)}$ = 14.09, p = .003.

To break down the interaction, the effect of PE on recognition was analyzed separately for correct and incorrect prediction outcomes. We compared each bin with the first one, to test whether increasingly higher PE significantly affected memory encoding. Results showed that for incorrect prediction outcomes, the difference between the first and the second was significant (β = - 0.0557, p_{corr} = .040, OR = 1.06). In addition, the comparisons between the third and the first, and the fourth and the first, were both significant, (p_{corr} < .001). For correct prediction outcomes, the comparison between first and second quantile, and first and third quantile did not reach significance (ps_{corr} >.114), whereas the comparison between the fourth and the first quantile was significant (β = 0.081, p_{corr} = .009, OR = 1.08). These results suggest that while a PE error generated by incorrect prediction impairs memory even when prior expectations are not very strong, for correct predictions a higher PE is needed in order to observe benefits for memory encoding.

Analysis of Choice-dependent PE and Memory

In addition to considering PE as depending on the category presented in each trial, we also decided to analyze the effects PE dependent on participants' choice on memory. Such PE is similar to the signed PE considered by previous studies (e.g., 3; 4) and thus allows for a direct comparison between the current study and the previous ones. A graph of the relationship between choice-dependent PE and recognition accuracy is presented in Figure S9. Merged data from Experiment 1 and 2 were submitted to a generalized linear mixed-effects model, in which choice-dependent PE, learning rate, experiment, and their interactions were added as fixed effects, participants were added as random intercept, and choice-dependent PE was added as random slope. Results revealed a main effect of choice-dependent PE ($\chi^2_{(1)}$ = 13.05, p < .001) with a positive linear relationship between choice-dependent PE and memory (β = 0.24, OR = 1.27). We then considered the positive and negative sides of the choice-dependent PE separately. For the positive side, the positive relationship between choice-dependent PE and memory was significant (β = 0.80, p < .001, OR = 2.22), showing that the stronger the choice-dependent positive PE, the better the subsequent memory. For the negative side, there was a significant relationship with negative choice-dependent PE (β = 0.94, p < .001,

OR = 2.56), showing that the more negative the choice-dependent PE, the worse was the subsequent memory.

Analysis of Memory as a Function of Subsequent Similar Scenes

Following suggestions from a reviewer, we tested the possibility that the the number of subsequent similar scenes that followed each encoded image could modulate encoding strength. The reason behind this is that high PE can be beneficial in the case of successful predictions because it can be useful for predicting similar subsequent trials. The relationship between the number of subsequent similar contexts, recognition memory, and prediction outcome is shown in Figure S10. This analysis showed that the main effects of number of subsequent scenes and prediction outcome, and their interaction, were not significant, ps $>.097$.

References

[1] Murphy, K. P. *Machine learning: A probabilistic perspective* (MIT Press, Cambridge, 2012).

[2] Sutton, R. S. & Barto, A. G. *Reinforcement Learning: An Introduction* (MIT Press, Cambridge, 1998).

[3] De Loof, E. *et al.* Signed reward prediction errors drive declarative learning. *PLoS One* **13**, e0189212 (2018).

[4] Jang, A. I., Nassar, M. R., Dillon, D. G. & Frank, M. J. Positive reward prediction errors during decision making strengthen memory encoding. *Nature Human Behaviour* **3**, 719–732 (2019). URL `http://dx.doi.org/10.1038/s41562-019-0597-3`.

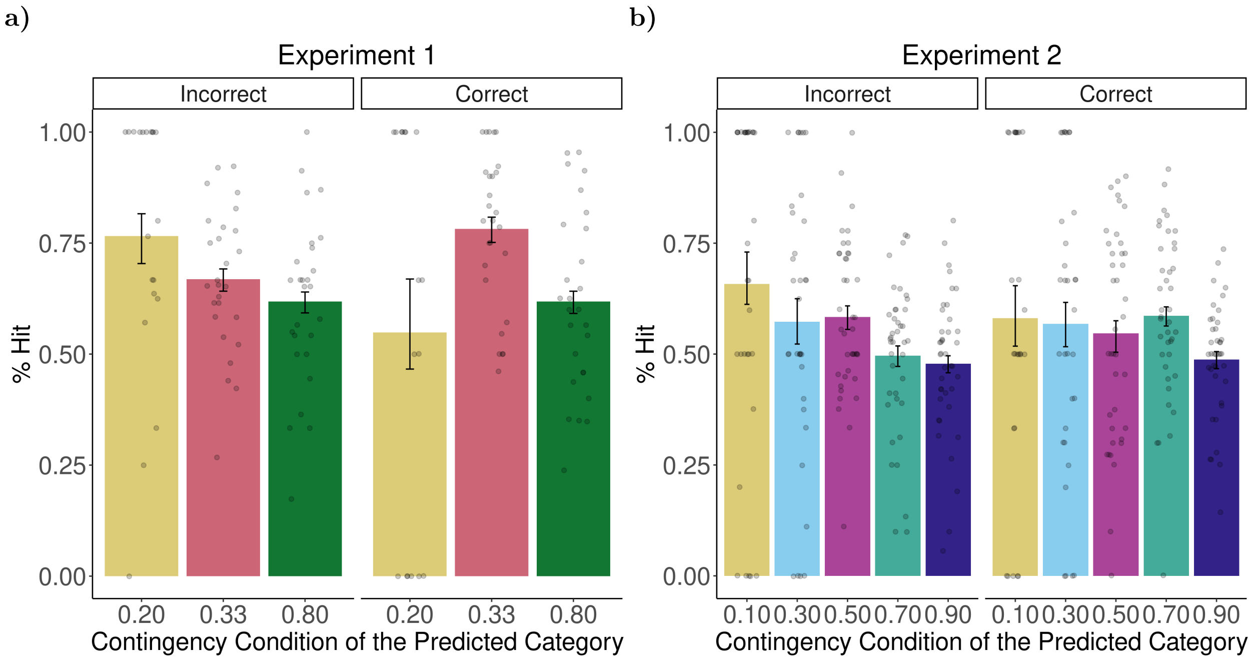

Figure S1: Recognition Memory as a Function of Contingency Condition of the Predicted Category and Prediction Outcome. Hit rates as a function of contingency condition of the predicted category and prediction outcome are shown for a) Experiment 1 and b) Experiment 2. Contingency condition of the predicted category represents the contingency condition related to the object category predicted by participants on a given trial. The dots represent the participants while the error bars show the standard error.

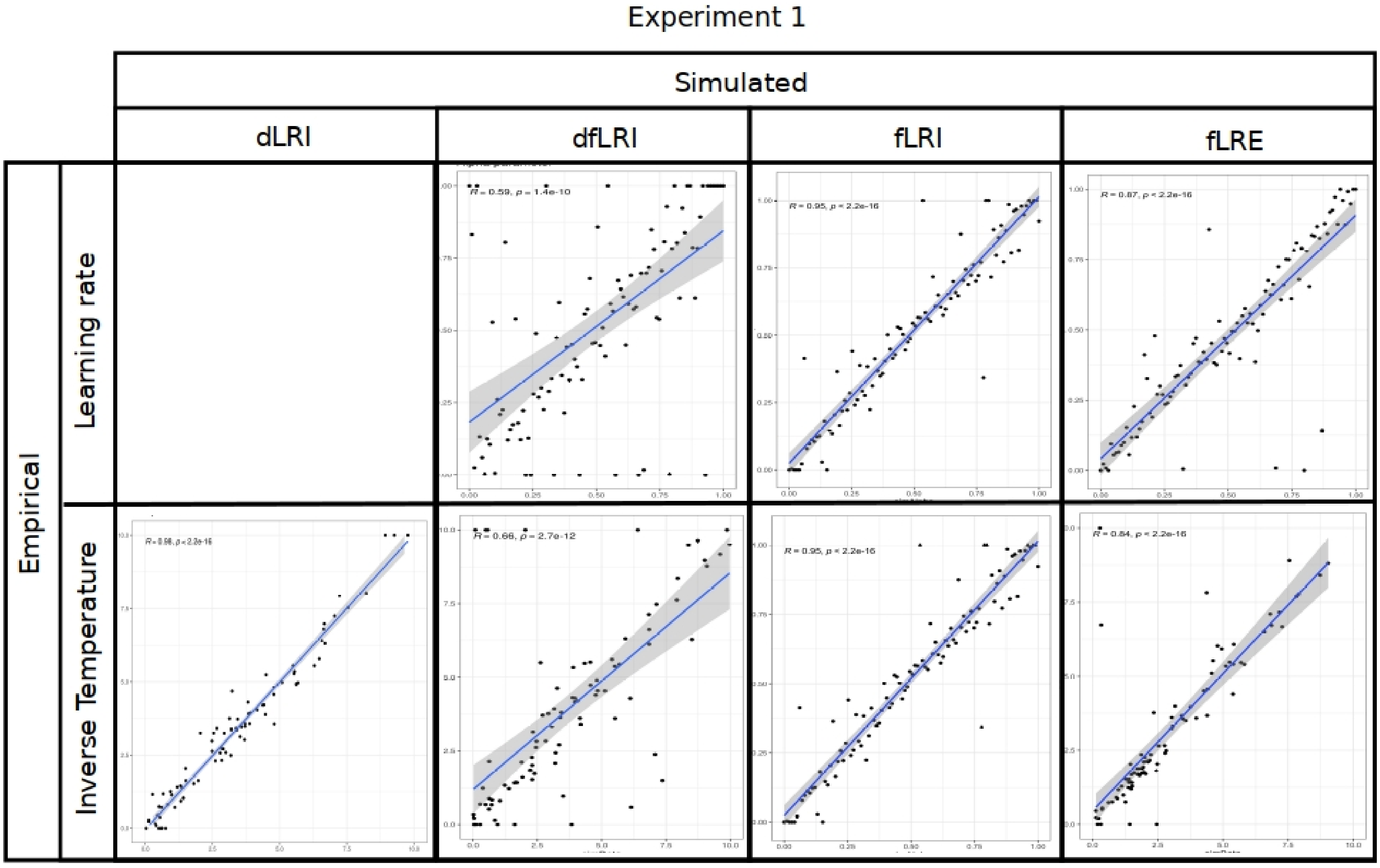

Figure S2: Parameter Recovery. Parameter Recovery for Experiment 1. Shadows show 95% confidence intervals.

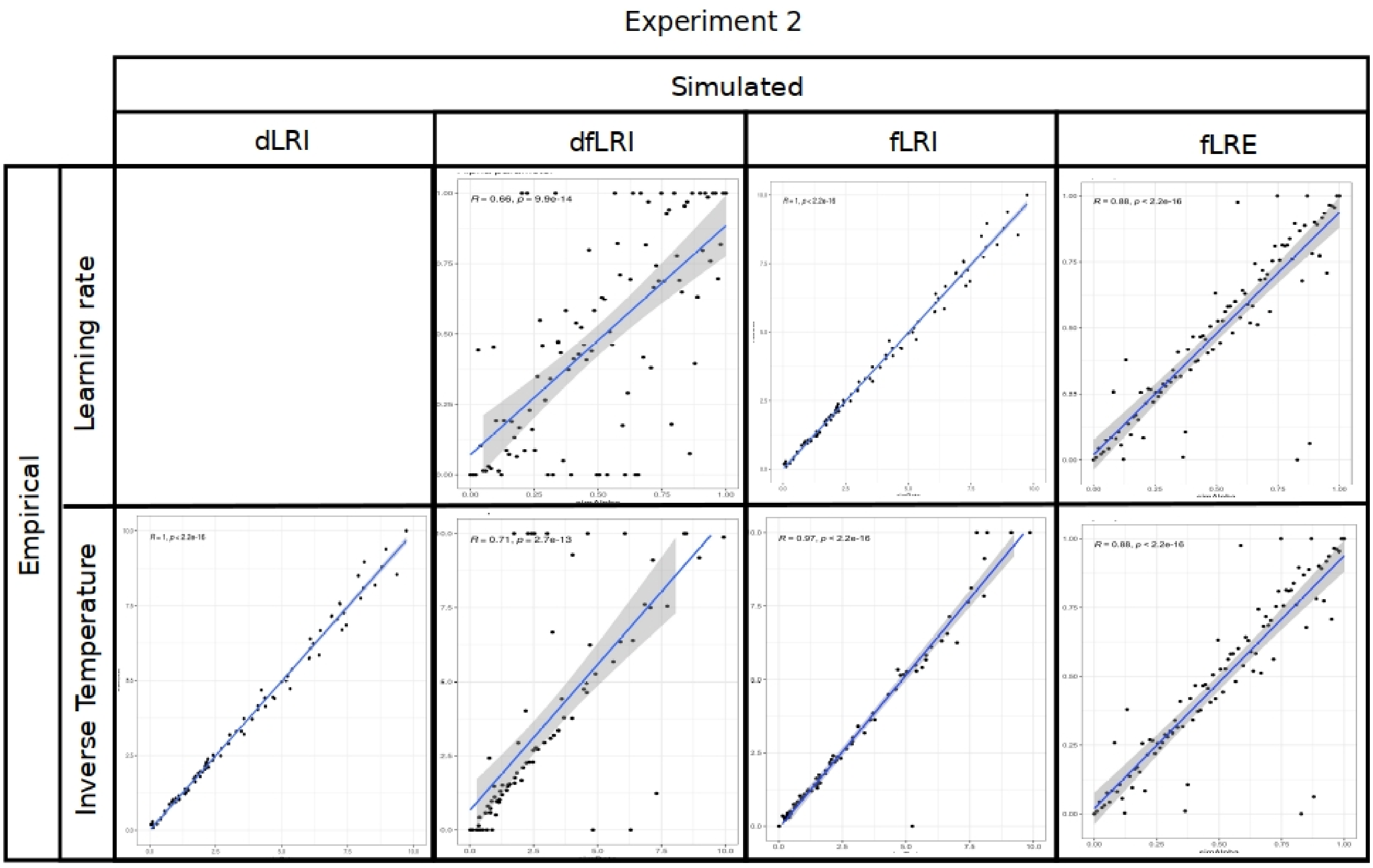

Figure S3: Parameter Recovery. Parameter Recovery for Experiment 2. Shadows show 95% confidence intervals.

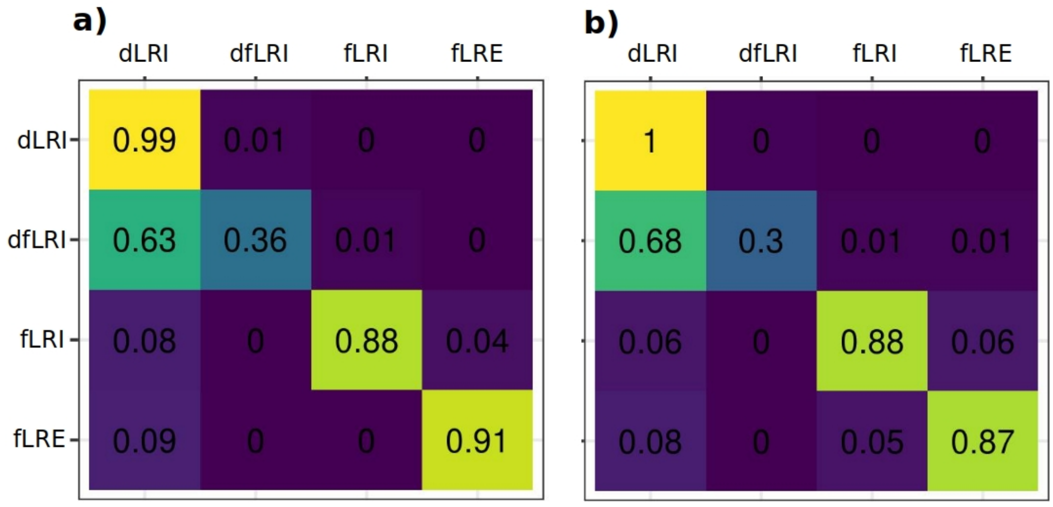

Figure S4: Model Recovery. Confusion Matrices showing model recovery for a) Experiment 1 and b) Experiment 2. The numbers show the probability of data generate by model X to be best fit by model Y.

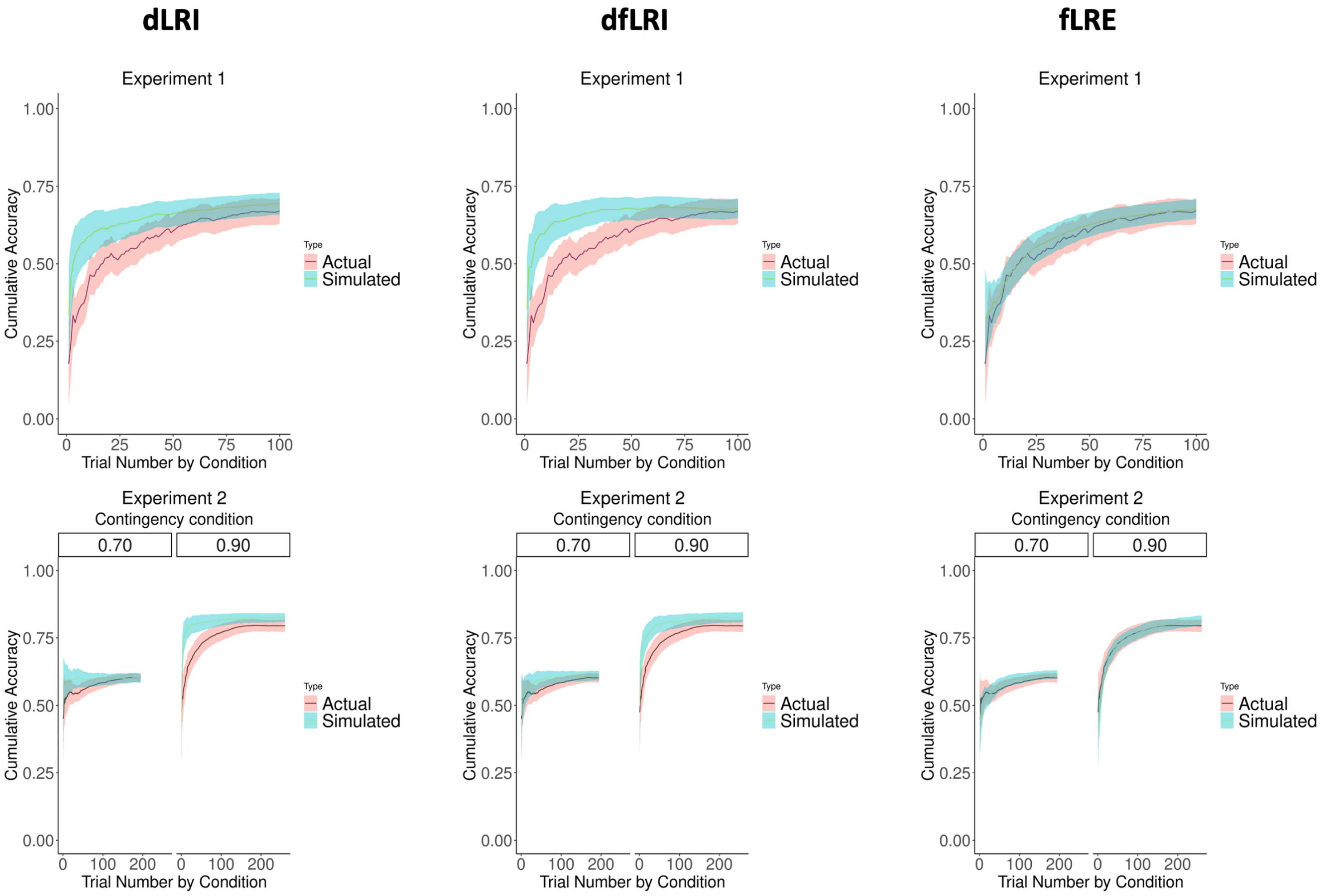

Figure S5: Simulated vs Empirical Data for the dLRI, dfLRI, and fLRE Models. Simulated data (green line) and actual data (red line) are overlapped, for Experiment 1 (top) and Experiment 2 (bottom).Coloured shadows show 95% confidence intervals.

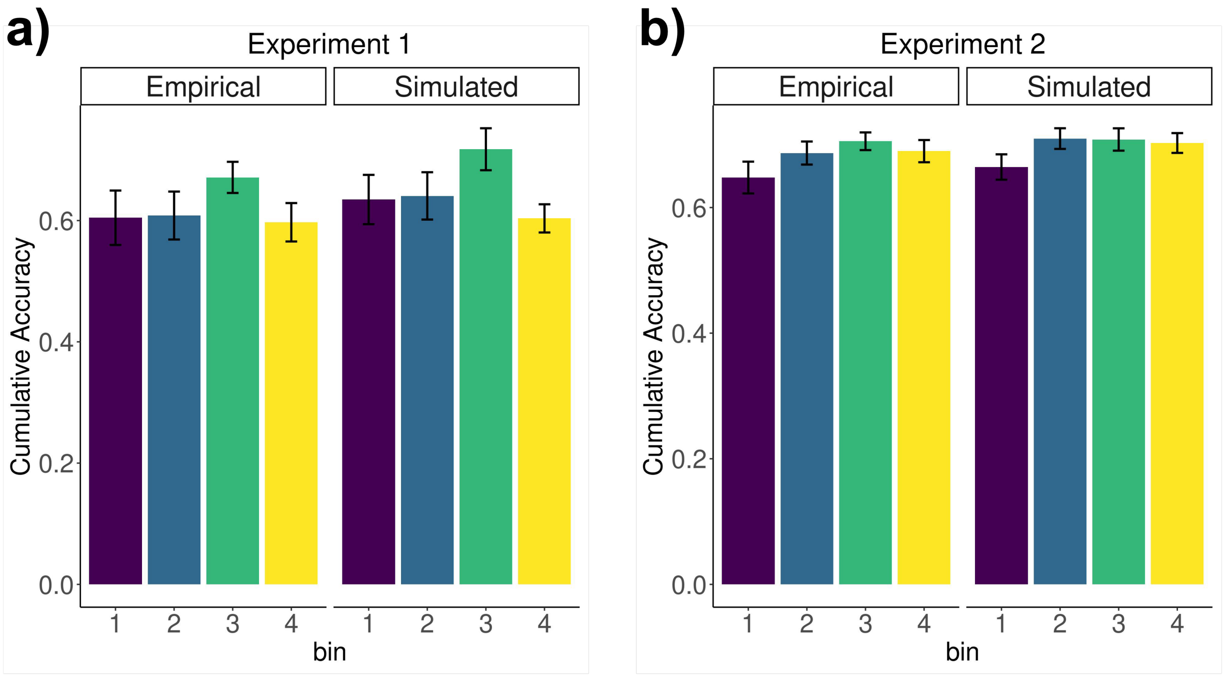

Figure S6: Learning Rate Validation. Cumulative accuracy on the learning and encoding task for simulated and actual data at different (binned) learning rates levels for a) Experiment 1 and b) Experiment 2.

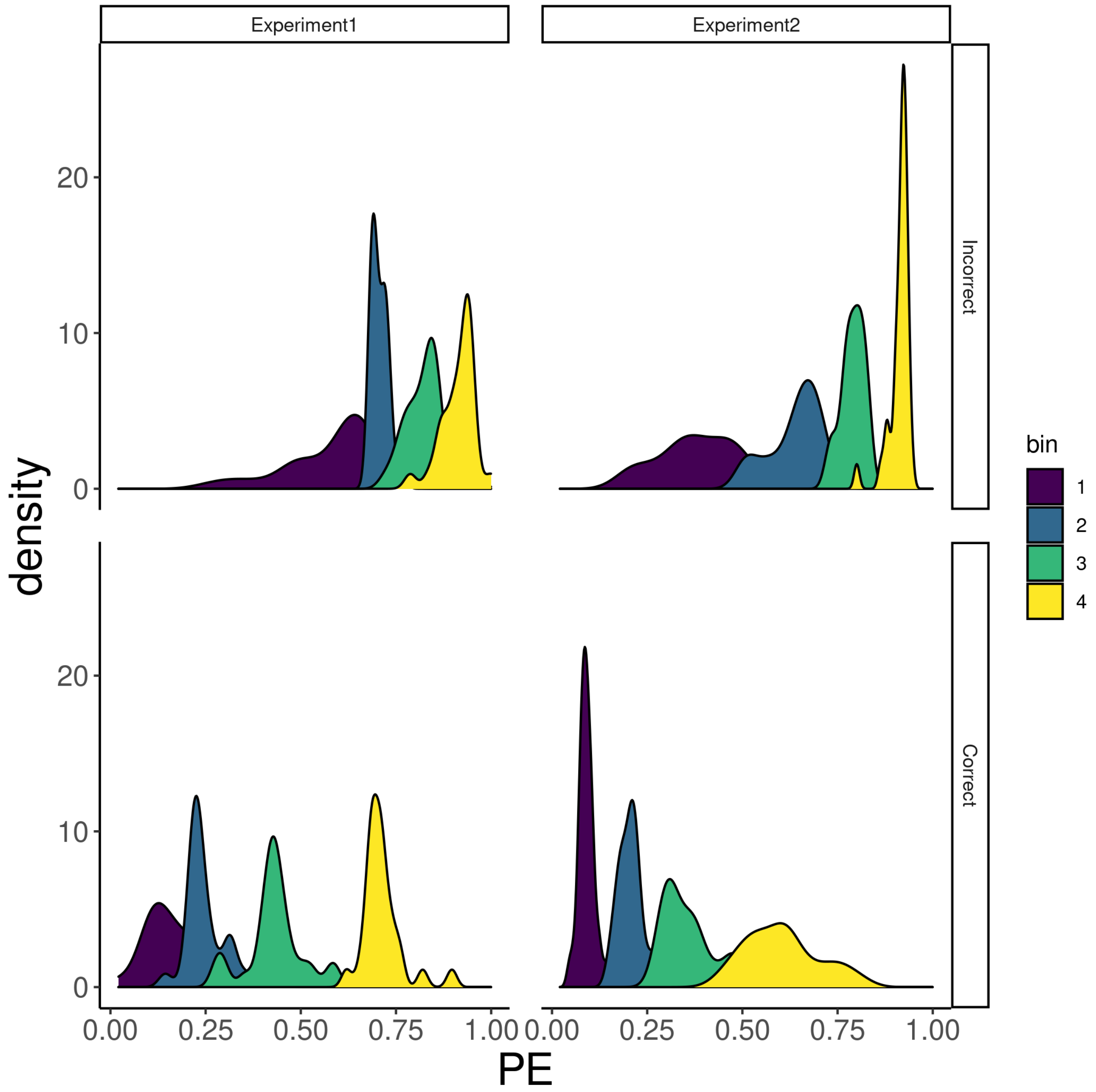

Figure S7: Distribution of Binned PE. Distribution of PE after binning it, as a function of prediction outcome and experiment.

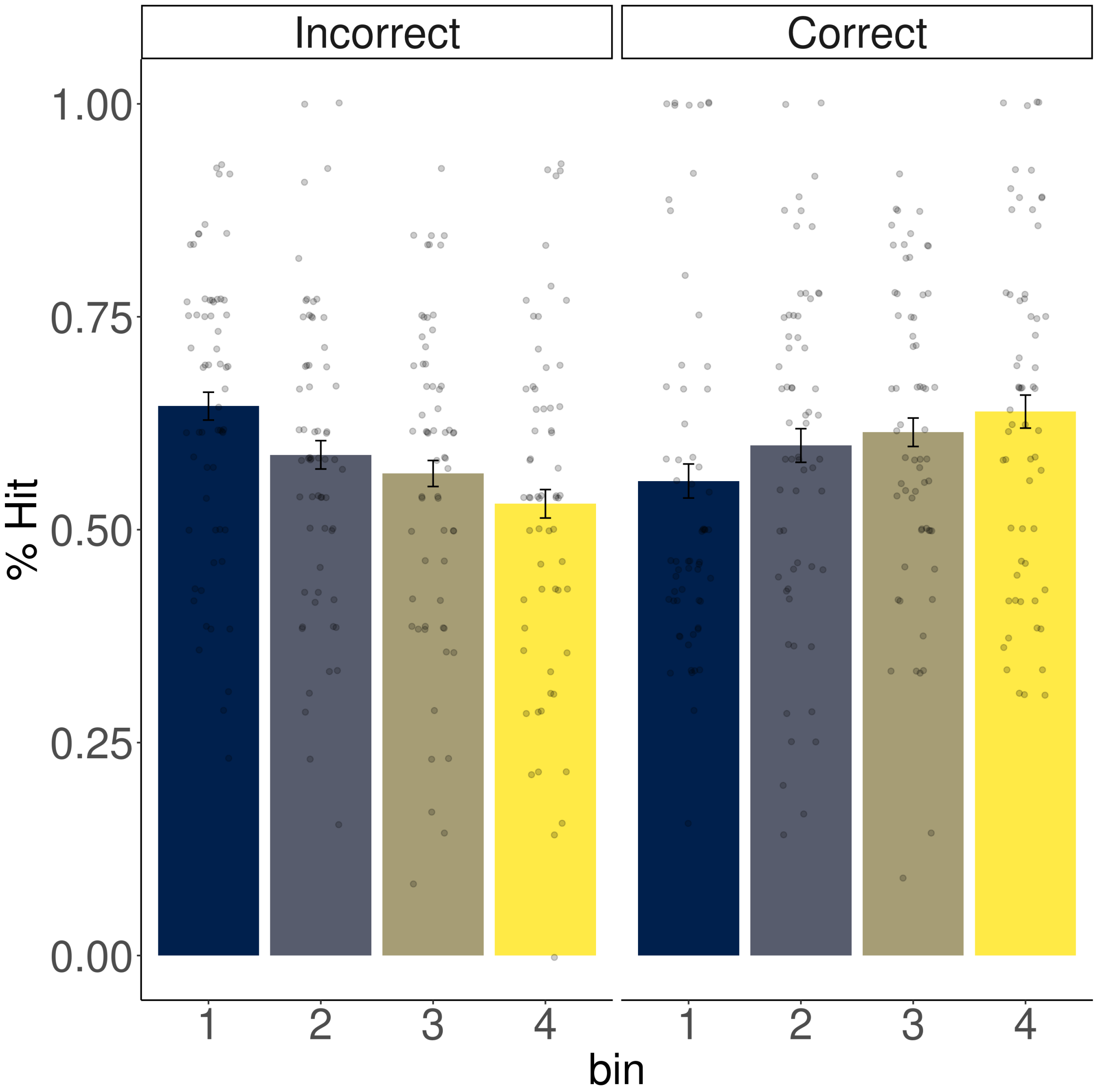

Figure S8: Hit Rate by Binned PE. Effect of binned PE on hit rate as a function of prediction outcome. Error bars represent the standard error.

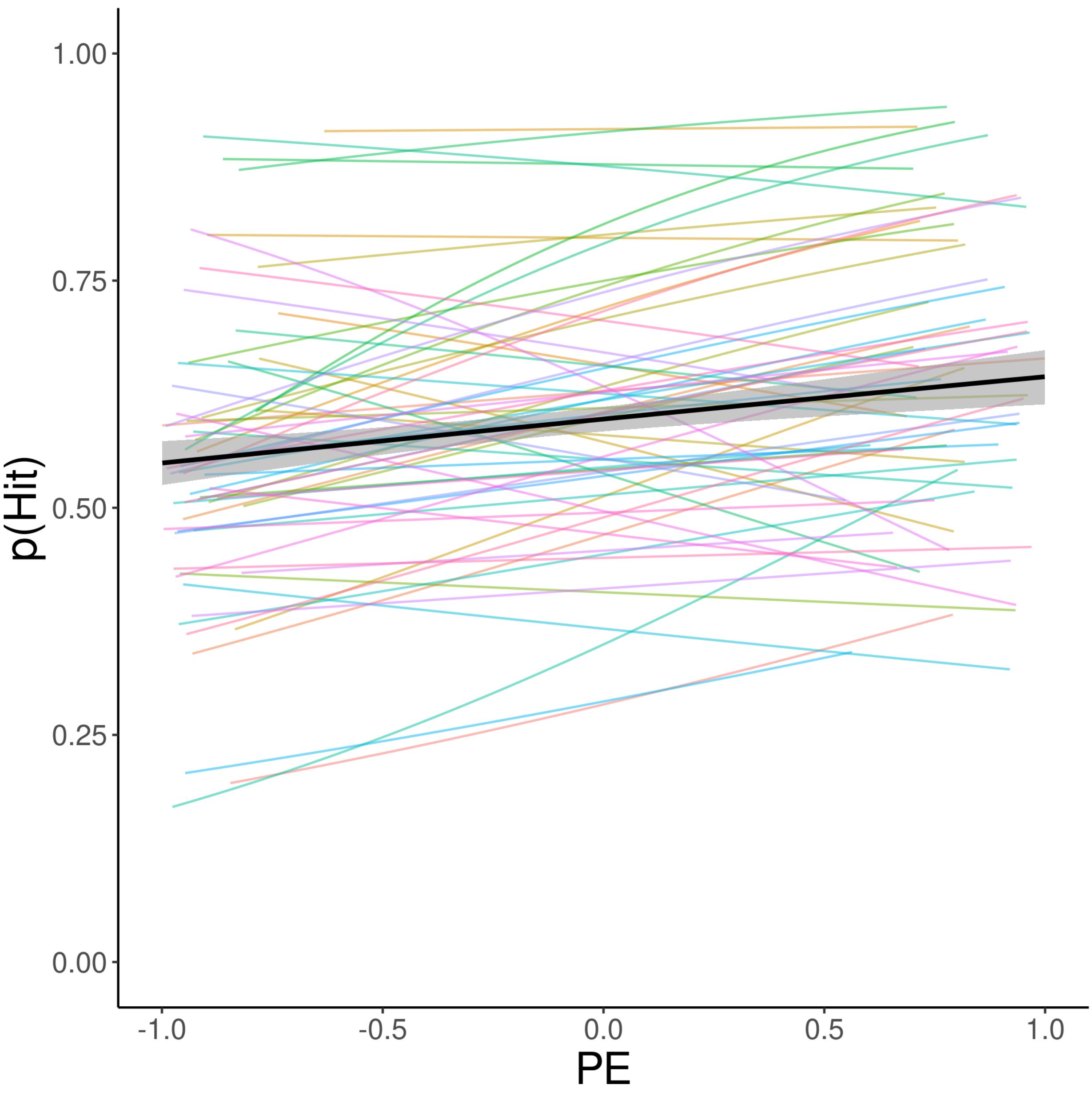

Figure S9: Computationally-derived Choice-dependent PE and Memory. Spaghetti plot of the relationship between model-derived choice-dependent PE and recognition memory. The shadow around the black line represents 95% confidence interval.

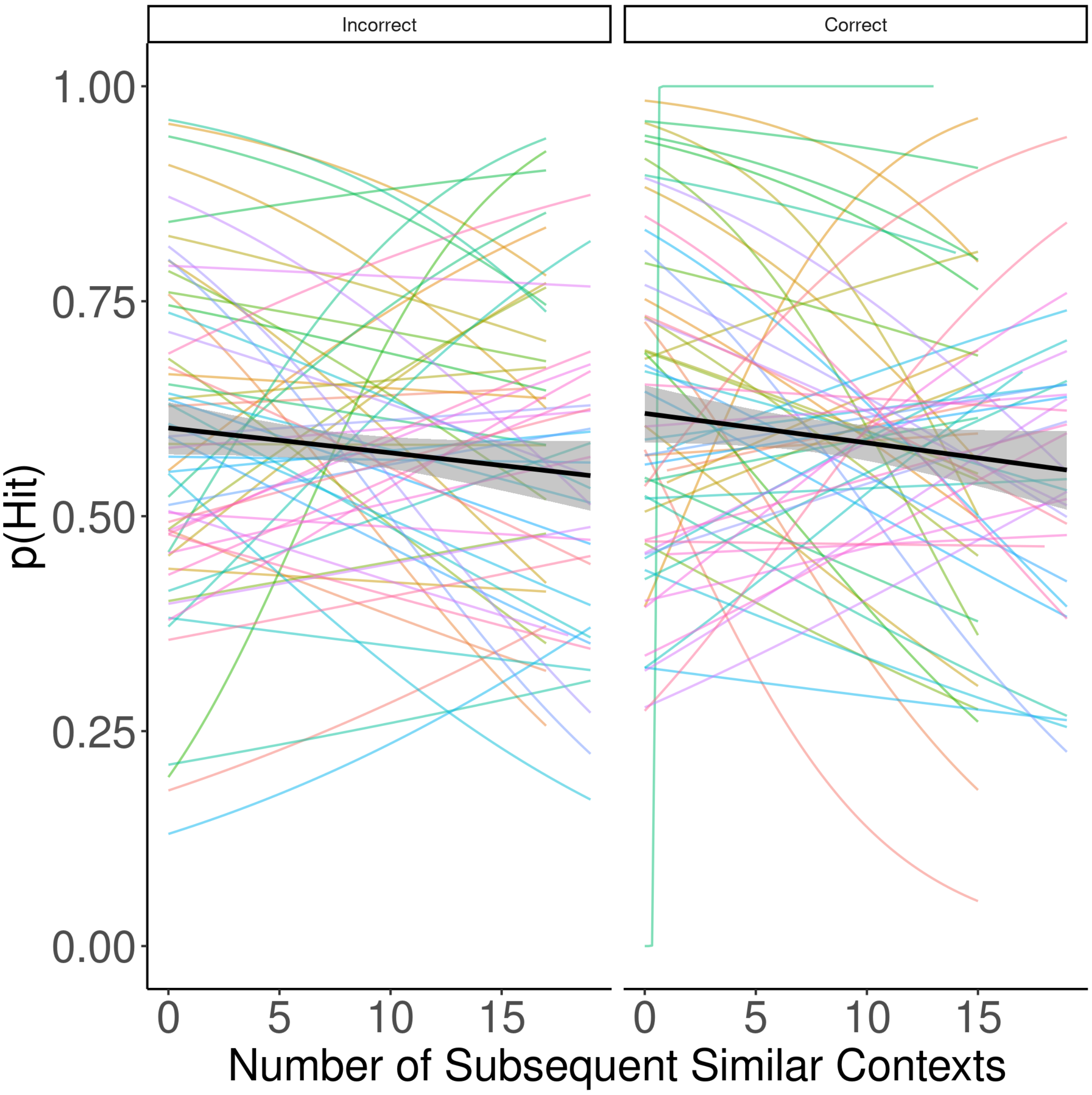

Figure S10: Recognition Memory as a Function of Number of Similar Subsequent Scenes and Prediction Outcome. Spaghetti plot of the relationship between the number of similar subsequent scenes of an item, prediction outcome, and recognition memory. The shadow around the black line represents 95% confidence interval.

Table S1

Experiment 1

Contingency learning phase

Prior condition	Object categ	Tokens	Repetitions	n_trials	%
**0.80-0.20 context 1**	Cat1	2	8	16	80
	Cat2	2	1	2	10
	Cat3	2	1	2	10
**0.80-0.20 context 2**	Cat1	2	1	2	10
	Cat2	2	8	16	80
	Cat3	2	1	2	10
**0.80-0.20 context 3**	Cat1	2	1	2	10
	Cat2	2	1	2	10
	Cat3	2	8	16	80
**0.33-0.33-0.33 context 1**	Cat1	2	1	2	33,33333333
	Cat2	2	1	2	33,33333333
	Cat3	2	1	2	33,33333333
**0.33-0.33-0.33 context 2**	Cat1	2	1	2	33,33333333
	Cat2	2	1	2	33,33333333
	Cat3	2	1	2	33,33333333
**0.33-0.33-0.33 context 3**	Cat1	2	1	2	33,33333333
	Cat2	2	1	2	33,33333333
	Cat3	2	1	2	33,33333333

Encoding phase

Prior condition	Object categ	Tokens	Repetitions	n_trials	%	%
**0.80-0.20 context**	Cat1	8	1	8	10	80
	Cat1fillers	8	7	56	70	
	Cat2	8	1	8	10	10
	Cat3	8	1	8	10	10
**0.80-0.20 context**	Cat1	8	1	8	10	10
	Cat2	8	1	8	10	80
	Cat2fillers	8	7	56	70	
	Cat3	8	1	8	10	10
**0.80-0.20 context**	Cat1	8	1	8	10	10
	Cat2	8	1	8	10	10
	Cat3	8	1	8	10	80
	Cat3fillers	8	7	56	70	
**0.33-0.33-0.33 co**	Cat1	8	1	8	33,333333	33,33333333
	Cat2	8	1	8	33,333333	33,33333333
	Cat3	8	1	8	33,333333	33,33333333
**0.33-0.33-0.33 co**	Cat1	8	1	8	33,333333	33,33333333
	Cat2	8	1	8	33,333333	33,33333333
	Cat3	8	1	8	33,333333	33,33333333
**0.33-0.33-0.33 co**	Cat2	8	1	8	33,333333	33,33333333
	Cat3	8	1	8	33,333333	33,33333333
	Cat1	8	1	8	33,333333	33,33333333

Retrieval phase

Prior condition	Object categ	Tokens	%
**0.80-0.20 context**	Cat1	8	33,333333
	Cat2	8	33,333333
	Cat3	8	33,333333
**0.80-0.20 context**	Cat1	8	33,333333
	Cat2	8	33,333333
	Cat3	8	33,333333
**0.80-0.20 context**	Cat1	8	33,333333
	Cat2	8	33,333333
	Cat3	8	33,333333
**0.33-0.33-0.33 co**	Cat1	8	33,333333
	Cat2	8	33,333333
	Cat3	8	33,333333
**0.33-0.33-0.33 co**	Cat1	8	33,333333
	Cat2	8	33,333333
	Cat3	8	33,333333
**0.33-0.33-0.33 co**	Cat1	8	33,333333
	Cat2	8	33,333333
	Cat3	8	33,333333
**Lures**	Cat1	48	33,333333
	Cat2	48	33,333333
	Cat3	48	33,333333

Experiment 2

Contingency learning phase

Prior condition	Object categ	Tokens	Repetitions	n_trials	%
**0.90-0.10 context 1**	Cat1	4	9	36	90
	Cat2	4	1	4	10
**0.90-0.10 context 2**	Cat1	4	1	4	10
	Cat2	4	9	36	90
**0.70-0.30 context 1**	Cat1	4	7	28	70
	Cat2	4	3	12	30
**0.70-0.30 context 2**	Cat1	4	3	12	30
	Cat2	4	7	28	70
**0.50-0.50 context 1**	Cat1	4	5	20	50
	Cat2	4	5	20	50
**0.50-0.50 context 2**	Cat1	4	5	20	50
	Cat2	4	5	20	50

Encoding phase

Prior condition	Object categ	Tokens	Repetitions	n_trials	%	%
**0.90-0.10 context**	Cat1	10	1	10	10	90
	Cat1fillers	5	16	80	80	
	Cat2	10	1	10	10	10
**0.90-0.10 context**	Cat1	10	1	10	10	10
	Cat2	10	1	10	10	90
	Cat2fillers	5	16	80	80	
**0.70-0.30 context**	Cat1	10	1	10	28	71
	Cat1fillers	5	3	15	42	
	Cat2	10	1	10	28	29
**0.70-0.30 context**	Cat1	10	1	10	28	29
	Cat2	10	1	10	28	71
	Cat2fillers	5	3	15	42	
**0.50-0.50 context**	Cat1	5	1	5	16,666667	50
	Cat1fillers	5	2	10	33,333333	
	Cat2	5	1	5	16,666667	50
	Cat2fillers	5	2	10	33,333333	
**0.50-0.50 context**	Cat1	5	1	5	16,666667	50
	Cat1fillers	5	2	10	33,333333	
	Cat2	5	1	5	16,666667	50
	Cat2fillers	5	2	10	33,333333	

Retrieval phase

Prior condition	Object categ	Tokens	%
**0.90-0.10 context**	Cat1	10	50
	Cat2	10	50
**0.90-0.10 context**	Cat1	10	50
	Cat2	10	50
**0.70-0.30 context**	Cat1	10	50
	Cat2	10	50
**0.70-0.30 context**	Cat1	10	50
	Cat2	10	50
**0.50-0.50 context**	Cat1	5	50
	Cat2	5	50
**0.50-0.50 context**	Cat1	5	50
	Cat2	5	50
**Lures**	Cat1	50	50
	Cat2	50	50
